# Barriers and facilitators of evidence-informed health policymaking in Nepal: A scoping review

**DOI:** 10.1371/journal.pone.0358730

**Published:** 2026-10-26

**Authors:** Padam Simkhada, Kaung Suu Lwin, Bikash Koirala, Akina Shrestha, Hridaya Devkota, Anju Vaidya, Bibha Simkhada

**Affiliations:** 1 Research and Innovation Office, University of Chester, Chester, United Kingdom; 2 School of Human and Health Sciences, University of Huddersfield, Huddersfield, United Kingdom; 3 Green Tara Nepal, Kathmandu, Nepal; 4 Department of Public Health, Kathmandu University School of Medical Sciences, Dhulikhel, Nepal; Group for Technical Assistance / Asian College for Advance Studies, Purbanchal University, NEPAL

## Abstract

**Background:**

Evidence-informed health policymaking (EIPM) is essential for strengthening health systems and achieving equitable health outcomes. Despite policy commitments in Nepal, the translation of research evidence into policy and practice remains limited. This scoping review synthesizes the barriers and facilitators influencing EIPM across health policy domains in Nepal.

**Methods:**

We conducted a scoping review following the Arksey and O’Malley framework and PRISMA-ScR guidelines. Peer-reviewed studies published between 2015 and 2025 that examined barriers and facilitators of EIPM in Nepal were identified through searches of PubMed, Scopus, Embase, and Global Health. Two reviewers independently screened studies, extracted data, and conducted descriptive and thematic analyses.

**Results:**

Of 2,139 records identified, 26 studies met the inclusion criteria. The studies covered maternal and neonatal health, non-communicable diseases (NCDs), nutrition, emergency care, health systems governance, climate-health, public health regulation, and COVID-19 ethics. Barriers were grouped into five themes: systemic and governance challenges; capacity and resource constraints; evidence-related issues; contextual and cultural factors; and operational gaps. Common barriers included political interference, fragmented coordination, workforce shortages, inadequate funding, donor-driven agendas, poor dissemination of research, and limited implementation guidance. Facilitators clustered into four themes: institutional and structural enablers, political and leadership commitment, capacity building and stakeholder engagement, and global and donor support. Sector-specific differences were observed, with socio-cultural barriers more prominent in maternal and neonatal health, governance and coordination challenges particularly evident in NCD policies, and technical capacity and data limitations more pronounced in climate-health policy.

**Conclusions:**

Nepal’s EIPM landscape reflects strong commitment but uneven institutionalization. Strengthening governance, institutional capacity, evidence systems, and coordination across federal, provincial, and local institutions is essential to improve the translation of research into policy and practice. Institutionalizing EIPM requires not only producing evidence but also creating systems that routinely generate, translate, and use evidence for decision-making.

## Introduction

Evidence-informed health policymaking (EIPM) is widely recognized as a cornerstone of effective, equitable, and accountable governance in health systems globally [1,2]. By systematically integrating research evidence, contextual data, and experiential knowledge into the policy cycle, from agenda setting to implementation and evaluation, EIPM enhances transparency, improves resource allocation, and strengthens health system performance [3]. The global movement toward EIPM has been reinforced by initiatives such as WHO’s health systems strengthening agenda and the Sustainable Development Goals, which emphasize evidence-based decision-making as critical for achieving universal health coverage [1–3].

In low- and middle-income countries (LMICs) such as Nepal, the imperative for EIPM is particularly acute. Nepal’s health system faces persistent resource constraints, growing population health needs, and the complexities introduced by federalization. National frameworks, including the *National Health Policy 2019* and the *Health Sector Strategic Plan 2023–2030*, explicitly call for embedding research evidence into policy processes [4,5]. Despite these commitments, the translation of evidence into actionable policy remains limited, creating a critical gap between knowledge generation and policy implementation [6]. This gap undermines the efficiency and equity of health interventions and weakens accountability mechanisms [7].

Several systemic and institutional barriers contribute to this gap. These include limited capacity among policymakers to access, appraise, and apply evidence; preference for anecdotal or politically expedient information; weak institutional mechanisms for knowledge translation; and inadequate dissemination of research in accessible formats [7,8]. Research findings are often poorly communicated, and fragmented engagement between researchers and policymakers perpetuates siloed practices, reducing opportunities for co-production of policy-relevant evidence [4,9]. Similar challenges have been documented across LMICs, where political cycles, donor influence, and competing priorities further constrain evidence use [7,9,10].

Conversely, multiple facilitators offer pathways to strengthen EIPM. Capacity-building initiatives for policymakers, structured policy dialogues, evidence briefs, and institutional platforms such as knowledge translation units have demonstrated potential to bridge the evidence–policy divide [10,11]. International experiences suggest that organizational factors such as leadership commitment, dedicated funding, and institutionalized processes, are critical for sustaining evidence use [11]. Nepal’s federal governance structure also presents opportunities for sub-national innovation, enabling context-specific adaptation of evidence-informed strategies [6].

Despite these developments, there is no comprehensive synthesis of the barriers and facilitators shaping EIPM in Nepal. A preliminary search of major databases and evidence synthesis platforms revealed no existing scoping or systematic reviews on this topic. Addressing this gap is essential for informing strategies that institutionalize evidence use and build resilient health systems. This scoping review therefore aims to systematically map the existing literature, identify critical challenges, highlight enabling factors, and propose actionable directions for strengthening EIPM in Nepal.

## Methods

### Study design

We conducted a scoping review to map existing evidence on barriers and facilitators of evidence-informed health policymaking (EIPM) in Nepal. The review followed the methodological framework proposed by Arksey and O’Malley [12], refined by Levac et al. [13], and the Joanna Briggs Institute (JBI) guidelines [14]. Reporting adhered to the PRISMA extension for scoping reviews (PRISMA-ScR) checklist (S1 File) [15].

### Eligibility criteria

Studies were included if they met the following criteria:

- **Population:** Health policymakers, researchers, and stakeholders in Nepal
- **Concept:** Barriers and facilitators between evidence generation and health policy implementation
- **Context:** Nepal’s health policy environment (institutions and organizations)
- **Types of Sources:** Peer-reviewed articles published from 2015 onwards in English or Nepali

Studies unrelated to Nepal, focused on clinical practice, or lacking analytical content were excluded.

### Information sources and search strategy

An electronic search was conducted in October 2025 through PubMed, Scopus, Embase, and Global Health using the keywords: evidence-informed, knowledge translation, policy making, barriers, challenges, facilitators, opportunities and Nepal. These keywords were searched in MEDLINE using the Boolean operators “AND” and “OR” (a detailed search strategy of each database is provided in S2 File).

### Study selection

All retrieved records were imported into Rayyan for reference management and duplicate removal. Following deduplication, two reviewers independently screened titles and abstracts against the predefined eligibility criteria. Records considered potentially relevant proceeded to full-text review. Full-text articles were independently assessed by the reviewers, and any disagreements were resolved through discussion and, where necessary, consultation with a third reviewer. Reasons for exclusion at the full-text screening stage were documented. The study selection process was conducted in accordance with PRISMA-ScR guidance and is illustrated in the PRISMA flow diagram (Fig 1).

**Fig 1:**
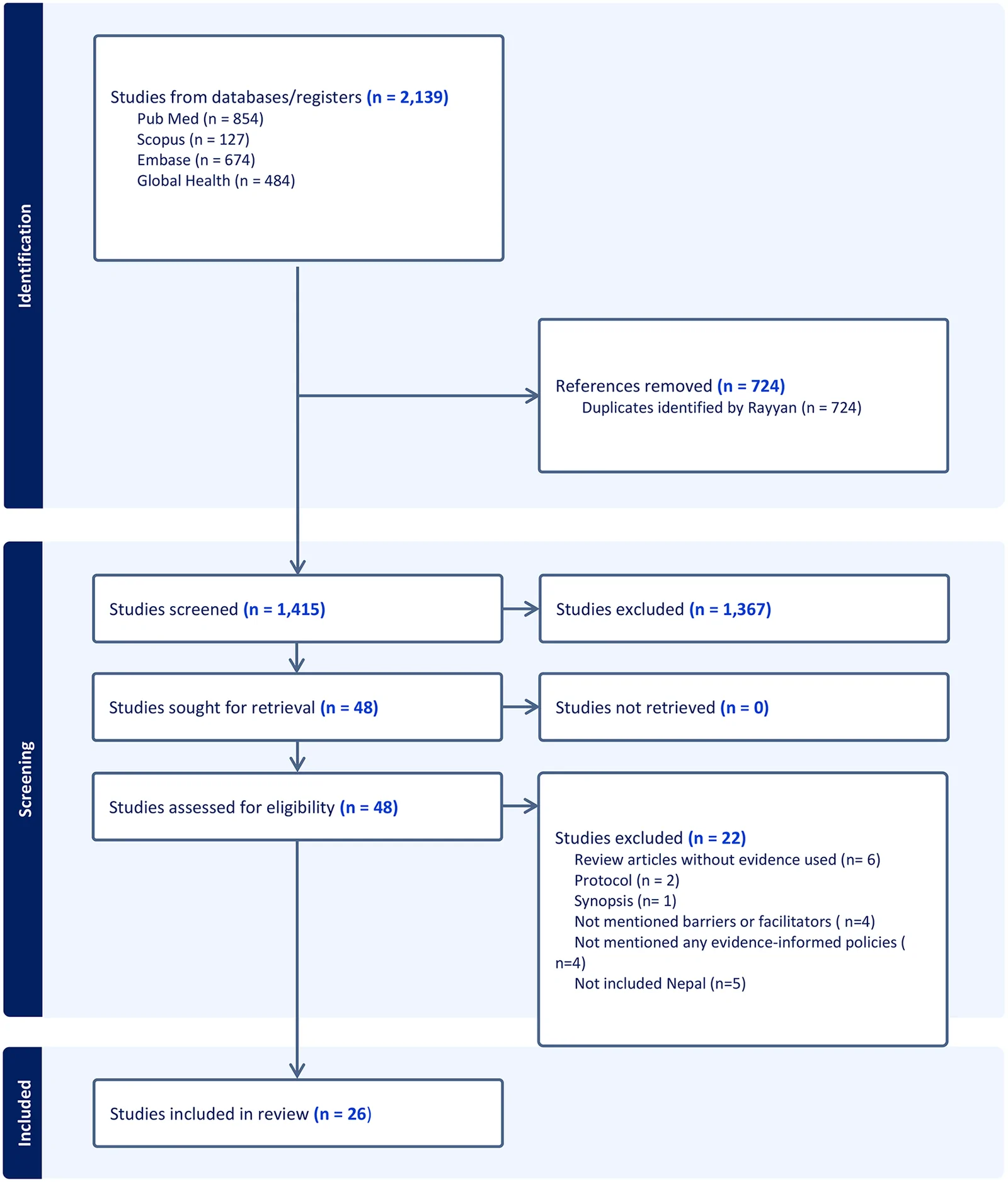
PRISMA flow diagram.

### Data extraction

Data were extracted using a structured Microsoft Excel tool based on the PCC framework (Population, Concept and Context). Extracted variables included:

- Study characteristics (authors, year, design)
- Population and setting
- Identified barriers and facilitators
- Institutional mechanisms and contextual factors
- Key findings and implications

Two reviewers independently extracted data, with disagreements resolved by consensus.

### Data analysis

We analysed the extracted data using both quantitative and qualitative approaches. Descriptive statistics were applied to summarize study characteristics, including publication year, study design, type of evidence, and source type.

For qualitative data, we conducted an inductive thematic synthesis. No predefined coding framework was applied; rather, codes were generated directly from the extracted data. Text segments related to barriers and facilitators were coded independently by two reviewers using a line-by-line approach. The reviewers compared coding outputs, and any discrepancies were resolved through discussion and consensus, with consultation from a third reviewer where necessary. Similar codes were subsequently grouped into descriptive categories, which were iteratively reviewed and refined to develop overarching themes reflecting systemic, institutional, and contextual factors influencing evidence-informed health policymaking in Nepal. The thematic synthesis followed an iterative process to ensure consistency, capture emerging patterns.

**Protocol and registration:** The review protocol was developed a priori but was not formally registered. To enhance transparency and accessibility, the protocol has been deposited in the Open Science Framework (OSF) and is available at: https://osf.io/fp27w.

**Ethics statement:** Ethical approval was not required because this scoping review used only existing, publicly available literature and did not involve human participants, individual-level identifiable data, or primary data collection.

## Results

A total of 2,139 records were identified through database searches. After the removal of 724 duplicate records, 1,415 records were screened by title and abstract. Following screening, 48 articles were retrieved and assessed for full-text eligibility. Of these, 22 articles were excluded for the following reasons: review articles without evidence used (n = 6), protocol papers (n = 2), synopsis article (n = 1), absence of barriers or facilitators relevant to the review objective (n = 4), no discussion of evidence-informed policies (n = 4), and studies not conducted in Nepal (n = 5). A total of 26 studies met the inclusion criteria and were included in the review. The complete study selection process is summarised in Fig 1. The highest number of studies was published in 2019 (n = 5), followed by 2020 (n = 4). The years 2016, 2021, 2022, 2023, and 2025 each contributed three studies, while 2015 and 2024 each contributed one study (Fig 2).

**Fig 2:**
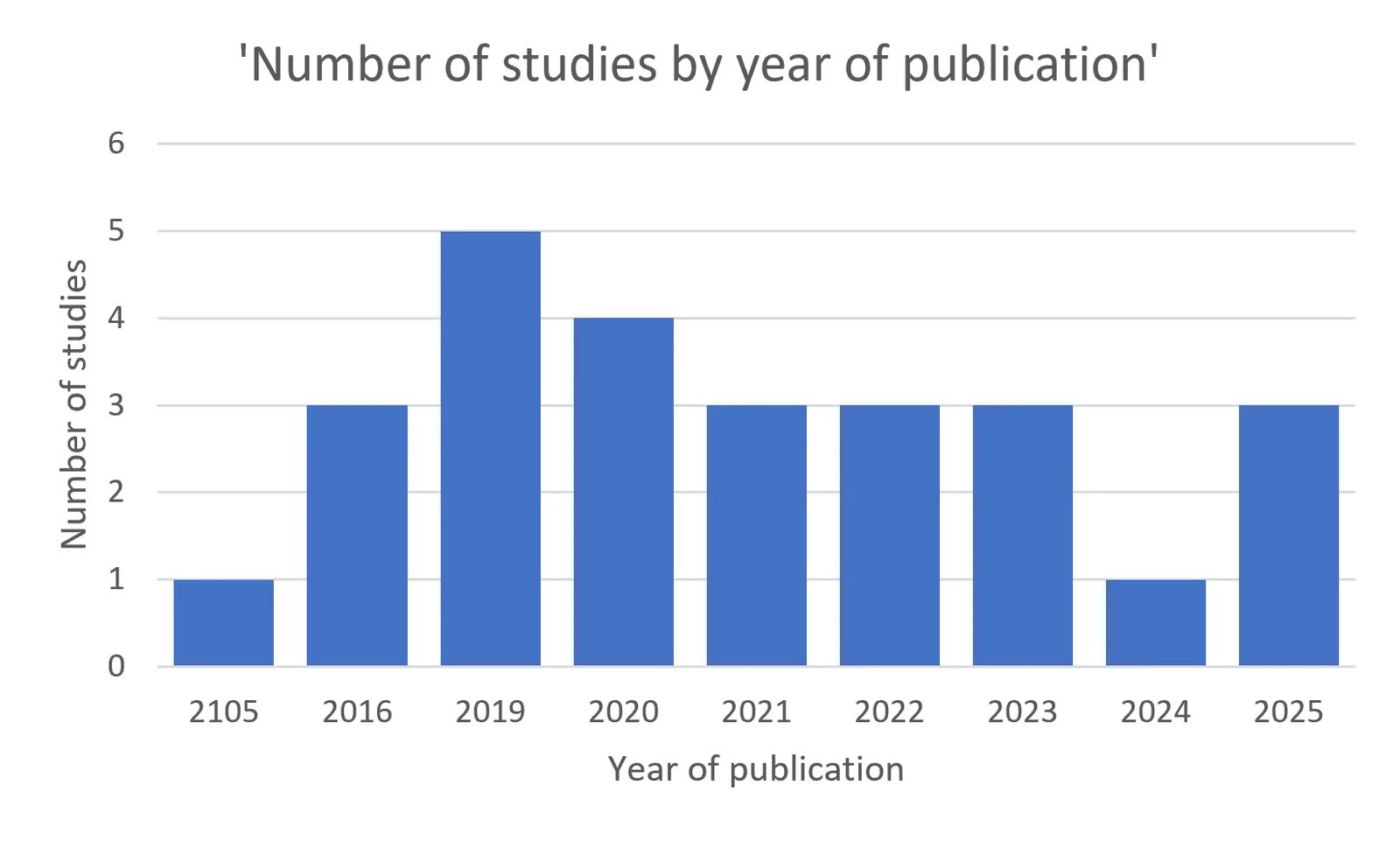
Number of studies by year of publication.

### Study characteristics

The included studies spanned 2015–2025, reflecting growing interest in evidence-informed health policymaking (EIPM) in Nepal. Study designs were predominantly qualitative (n = 18), complemented by mixed-methods studies (n = 5), policy document reviews (n = 4), and quantitative surveys (n = 3). The details information can be seen in S1 Table. As some studies were classified into more than one study design category, the totals exceed the 26 studies included in the review. Data collection approaches included key informant interviews (KIIs), focus group discussions (FGDs), policy document reviews, and secondary analyses of routine data. Analytical techniques ranged from thematic analysis and framework approaches to political economy analysis, SWOT, and Delphi methods.

Stakeholders represented a broad spectrum: government officials, policymakers, health workers, researchers, development partners, and civil society actors. Research settings were primarily the national health policy environment, with thematic foci in:

Maternal and neonatal health (MNH) [16–20]
Non-communicable diseases (NCDs) [21–25]
Nutrition and food security [26–28]
Emergency care [29]
Health systems and governance [30–35]
Public health regulation [36]
Climate and health [37,38]
COVID‑19 clinical ethics [39]

### Types and uses of evidence

Evidence sources included:

- National policies and strategic frameworks: Safe Motherhood Plan, WHO Package of Essential Noncommunicable Disease Interventions (PEN) program, Multi-sectoral Action Plan [16,22,23,25].
- Global guidelines: WHO “Best Buys” for NCDs, WHO-INTEGRATE Framework [21,35]
- Primary research and consensus exercises: Surveys, interviews, expert opinions [18,23,24,29]

Evidence use manifested as:

- Policy content alignment: Integration of WHO recommendations into national strategies [19,20,30]
- Program design and rollout: Implementation of PEN interventions for NCDs [22–25]
- Research prioritization: Structured exercises to identify emergency care priorities [29]
- Implementation adaptations: Use of mobile tools and community health workers to improve maternal health service delivery [18].

Most included studies were conducted exclusively in Nepal, while a smaller number (n = 6) were multi-country studies that included Nepal as one of the country cases. Findings from both study types contributed to the thematic synthesis.

### Barriers to evidence-informed policymaking

Barriers were pervasive across governance, capacity, evidence systems, and contextual domains. Five major themes emerged (Table 1):

**Table 1:** Synthesized barriers to evidence-informed policymaking in Nepal.

Theme	Representative Findings	References
Systemic and Governance Challenges	Political interference; corruption; weak accountability; fragmented governance; unclear roles across federal, provincial, local levels; poor intersectoral coordination	Paudel et al., 2019 [16]; Hipgrave et al., 2019 [17]; Buse et al., 2020 [21]; Namirembe et al., 2022 [26]; Witter et al., 2019 [30]; Dhimal et al., 2016 [31]
Capacity and Resource Constraints	Human resource shortages; inadequate budgets; weak digital governance; time constraints for evidence review; underdeveloped HIS	Hipgrave et al., 2019 [17]; Buse et al., 2020 [21]; Dhimal et al., 2016 [31]; Wasti et al., 2023 [32]; Khanal et al., 2025 [37]
Evidence-Related Issues	Low availability of priority-based research; donor-driven agendas; concerns about validity and generalizability; weak dissemination; preference for anecdotal evidence	Hipgrave et al., 2019 [17]; Buse et al., 2020 [21]; Wasti et al., 2023 [32]; Stratil et al., 2022 [35]
Contextual and Cultural Factors	Socio-cultural acceptability; geographic diversity; political uncertainty; frequent leadership changes; conflicting priorities	Paudel et al., 2019 [16]; Tiwari et al., 2023 [18]; Binagwaho et al., 2024 [20]; Namirembe et al., 2022 [26]; Stratil et al., 2022 [35]; Fisher et al., 2021 [36]
Operational Gaps	Infrastructure deficits; poor logistics; lack of operational detail in policy documents; weak implementation capacity; absence of monitoring frameworks	Witter et al., 2019 [30]; Dhimal et al., 2016 [31]; Fisher et al., 2021 [36]; Lamichhane Dhimal et al., 2025 [38]

1. **Systemic and Governance Challenges**

Governance-related barriers were among the most frequently cited. Studies reported political interference, corruption, and weak accountability mechanisms as critical impediments to evidence use [16,19,20,24,26]. Policy decisions were often influenced by partisan interests or donor priorities rather than empirical evidence. Fragmented governance structures and unclear roles among federal, provincial, and local actors created duplication and inefficiencies [20,24,25]. For example, Witter et al. (2019) highlighted how overlapping mandates between ministries and development partners led to conflicting advice and slowed policy implementation [30].

Poor intersectoral coordination—especially between health, finance, and social sectors—limited integrated responses to complex health challenges [19,21,32]. Decentralization, while promising, was often poorly implemented, resulting in weak institutionalization of policies and uneven capacity at subnational levels [17,19]. This lack of clarity in roles and responsibilities undermined accountability and created bottlenecks in translating evidence into actionable strategies [17,19,24].

2. **Capacity and Resource Constraints**

Limited human resources, inadequate budget allocations, and weak digital governance were recurrent barriers [19,20,28]. Policymakers faced time constraints for reviewing lengthy reports, reducing opportunities for evidence uptake [26]. Health information systems were underdeveloped, hindering timely data access and use [25,26]. Infrastructure gaps and logistical challenges further constrained operationalization of evidence-informed strategies [19,20]. For instance, Khanal et al. (2025) noted that resource limitations—both financial and human—hampered efforts to integrate climate change considerations into health policy [37].

3. **Evidence-Related Issues**

Several studies reported low availability of priority-based research, with outputs often donor-driven or misaligned with national needs [19,20,25]. Concerns over quality, validity, and generalizability of local research undermined trust among policymakers [20]. Dissemination practices were weak*—*evidence was rarely packaged into actionable formats such as policy briefs or one-pagers [20]. Preference for anecdotal evidence and political expediency over rigorous research persisted [17,18,29]. Dhimal et al. (2016) emphasized that research findings were often too micro-level or lacked specific recommendations, making them impractical for policy use [31].

4. **Contextual and Cultural Factors**

Socio-cultural norms and geographic diversity posed challenges to policy adaptation [16,31]. Political uncertainty and frequent leadership changes disrupted continuity in evidence use [18,27]. Conflicting priorities among stakeholders—such as balancing donor agendas with local needs—further complicated decision-making [17,18,29]. For example, Fisher et al. (2021) documented how private sector interference and lack of international actor support weakened regulatory efforts in public health [36].

5. **Operational Gaps**

Infrastructure deficits, poor logistics, and lack of clear operational details in policy documents were common [24,26]. Weak implementation capacity and absence of monitoring frameworks limited translation of evidence into practice [27]. Sapkota et al. (2022) noted that fragmented governance and workforce shortages hindered the rollout of NCD interventions despite strong political commitments [22].

### Facilitators of evidence use

Facilitators clustered into four categories, offering actionable pathways for strengthening EIPM (Table 2):

**Table 2:** Synthesized facilitators of evidence-informed policymaking in Nepal.

Theme	Representative Findings	References
Institutional and Structural Enablers	Evidence synthesis units (e.g., NHRC); digital libraries; decentralization with local health committees; strengthened HIS; intersectoral platforms	Dhimal et al., 2016 [31]; Lamichhane Dhimal et al., 2025 [38]
Political and Leadership Commitment	Strong political will; regulatory quality; accountability measures; alignment with SDGs and WHO frameworks	Hipgrave et al., 2019 [17]; Buse et al., 2020 [21]; Fisher et al., 2021 [36]; Lamichhane Dhimal et al., 2025 [38]
Capacity Building and Engagement	Regular researcher–policymaker dialogues; collaborative networks; structured prioritization exercises; stakeholder consultations; community empowerment	Tiwari et al., 2023 [18]; Sapkota et al., 2022 [22]; Namirembe et al., 2022 [26]; Lecky et al., 2020 [29]
Global and Donor Support	Consistent donor funding; WHO technical assistance; adaptation of global guidelines (WHO Best Buys, WHO-INTEGRATE); cross-country learning	Paudel et al., 2019 [16]; Binagwaho et al., 2024 [20], Buse et al., 2020 [21]; Stratil et al., 2022 [35]; Fisher et al., 2021 [36]

1. **Institutional and Structural Enablers**

Establishing evidence synthesis units (e.g., at NHRC) and digital libraries emerged as key recommendations [19,32]. These mechanisms can streamline access to research and produce actionable messages for policymakers. Decentralization, when coupled with local health committees and strengthened health information systems, supported context-specific decision-making [19]. Intersectoral platforms and formalized coordination mechanisms enhanced policy coherence [16,19,20]. For instance, the creation of multi-sectoral NCD committees facilitated integrated approaches to chronic disease prevention [16,19].

2. **Political and Leadership Commitment**

Strong political will, regulatory quality, and accountability measures were cited as critical enablers [19,20]. National priorities aligned with global commitments such as SDGs and WHO frameworks created momentum for evidence use [19,27]. Hipgrave et al. (2019) observed that political commitment, combined with well-informed media coverage, drove maternal and child health reforms despite resource constraints [17].

3. **Capacity Building and Engagement**

Regular researcher–policymaker dialogues, collaborative networks, and structured prioritization exercises facilitated mutual understanding and trust [20,22,23]. Stakeholder consultations and community empowerment initiatives strengthened legitimacy and uptake of evidence-informed policies [18,19]. Lecky et al. (2020) demonstrated how structured prioritization exercises in emergency care fostered inclusivity and contextual leadership, ensuring that research agendas aligned with policy needs [29].

4. **Global and Donor Support**

Consistent donor funding and technical assistance from WHO and other partners enabled adoption of global best practices [19,27]. Cross-country learning and adaptation of international guidelines (e.g., WHO “Best Buys,” WHO-INTEGRATE) were instrumental in shaping policy frameworks [21,35]. Binagwaho et al. (2024) highlighted how global implementation tools and donor coordination strengthened health systems and promoted accountability in under-five mortality reduction strategies [20].

### Policy area-specific patterns in barriers and facilitators

While several barriers and facilitators were observed across policy areas, some were more pronounced within specific health policy domains. In maternal and neonatal health, socio-cultural norms, limited community participation, weak decentralisation, and challenges in contextualising interventions were frequently identified as barriers to evidence-informed [16–20]. Key facilitators included community empowerment, local health committees, decentralised decision-making, and evidence-based contextual planning [16–20].

In non-communicable disease (NCD) policy, governance and implementation challenges were particularly prominent. Fragmented governance structures, weak accountability mechanisms, workforce shortages, financing constraints, and inadequate logistics were commonly reported, while political commitment, multisectoral coordination mechanisms, implementation of the WHO Package of Essential Noncommunicable Disease Interventions (PEN), and technical support from international partners facilitated evidence use in policymaking [21–25].

Nutrition-related policy studies highlighted weak multisectoral coordination, limited stakeholder ownership, resource disparities, and short-term funding arrangements as key barriers [26–28]. Facilitators included high-level advocacy, multisectoral nutrition platforms, local leadership, and capacity-building initiatives that supported cross-sectoral action [26–28].

In climate-health policy, knowledge gaps, limited availability and use of local data, insufficient technical capacity, inadequate funding, and weak intersectoral collaboration emerged as major challenges. Facilitators included political leadership, federal governance structures, cross-sectoral partnerships, and support from global actors and international policy frameworks [37,38].

## Discussion

This scoping review synthesizes barriers and facilitators of evidence-informed health policymaking (EIPM) in Nepal across domains including maternal and newborn health, non-communicable diseases (NCDs), nutrition, emergency care, health systems and governance, climate–health, public health regulation, and clinical ethics. The findings indicate a dynamic interplay among governance arrangements, institutional capacity, evidence ecosystems, and contextual factors. While political interference, fragmented coordination, resource constraints, and limited dissemination consistently impede evidence use [19,20,24,26], multiple facilitators, such as formal evidence functions, political commitments, structured engagement, and adaptation of global guidance, offer tangible avenues to strengthen EIPM [19,23,25,31].

Governance and accountability emerged as pivotal. Studies document how policy decisions can be swayed by partisan interests or external agendas, weakening routine use of research in decision-making [19,20,24]. Overlapping mandates and unclear responsibilities across federal, provincial, and local levels generate duplication and inefficiencies that stall policy translation [24]. Conversely, when leadership commitment is explicit, anchored by national priorities and supported by media and stakeholder alignment, evidence can catalyze reform even under constrained resources [17,19].

Institutional capacity and resourcing were recurrent constraints. Shortages of skilled staff, limited budgetary space, and underdeveloped digital governance reduced the absorptive capacity of institutions to appraise and apply evidence [17,26]. Policymakers’ time constraints, coupled with a scarcity of concise, policy-ready products, further diminished uptake [20,25]. Targeted investments in evidence brokering, analytic capacity, and interoperable health information systems (HIS) are likely to yield benefits, especially when paired with incentives and accountability for evidence use [18,26].

Challenges within the evidence ecosystem itself were salient. Several studies highlight misalignment of research agendas with policy priorities, donor-driven focus, and concerns about validity, generalizability, and credibility of local evidence [19,20,25]. Dissemination pathways were weak—few policy briefs or shorter formats tailored to time-pressed decision-makers—prompting reliance on anecdote or expediency [20]. Structured prioritization exercises and co-production approaches can help realign research with the felt needs of policymakers and implementers [18,23].

Contextual and cultural factors complicate policy design and implementation. Socio-cultural acceptability and Nepal’s geographic diversity challenge uniform adaptation of policies, especially in MNH and service delivery [16]. Political uncertainty and frequent leadership changes undermine continuity in evidence use and regulatory actions [18,27]. In regulatory arenas such as food marketing restrictions, private sector interference and limited national data for design were documented, constraining enactment and enforcement [36].

Operational gaps, including insufficient logistics, financing, and implementation detail, limit the pathway from policy intent to practice. Even where policies align with global best practice, absence of step-by-step operational guidance (roles, timelines, indicators, budgets, risk mitigation) impedes uptake [24,26]. Linking policy content to implementation frameworks (e.g., RE-AIM) and publishing playbooks for priority programs (e.g., integrated CHW models) improved fidelity and scalability in specific maternal health contexts [18].

An important finding of this review is that barriers and facilitators varied across policy domains. In maternal and neonatal health, evidence use was particularly influenced by socio-cultural norms, community participation, and the need for context-specific policy implementation [16–20]. In contrast, studies on non-communicable diseases primarily highlighted governance, financing, workforce, and coordination challenges, reflecting the multisectoral nature of NCD prevention and control [21–25]. Nutrition and climate-health policies relied heavily on multisectoral collaboration and external support, while climate-health studies additionally emphasized technical capacity, data limitations, and evidence gaps [26–28,37,38]. These findings suggest that although strengthening governance, evidence generation, and knowledge translation is important across all policy areas, tailored approaches are needed to address sector-specific challenges and opportunities.

### Policy implications

Implications for policy and practice follow directly from the findings. First, evidence-informed policymaking (EIPM) should be institutionalised through the establishment or strengthening of national and subnational evidence synthesis units that can translate research into policy briefs, options papers, economic analyses, and implementation guidance [25]. Second, governance arrangements for EIPM should be strengthened by clarifying roles, mandates, and coordination mechanisms across sectors and levels of government, particularly for multisectoral policy areas such as non-communicable diseases (NCDs), nutrition, and climate-health [21,24,28]. Third, investments are needed in technical capacity and digital infrastructure, including training policymakers in evidence appraisal and equity assessment, strengthening health information systems, and promoting concise and accessible evidence products for decision-makers [20,26].

Fourth, research agendas should be aligned more closely with national and local policy priorities through structured priority-setting processes involving policymakers, implementers, researchers, and communities. Publicly funded research should be encouraged to demonstrate policy relevance and practical applicability, while co-funding approaches may help reduce dependence on donor-driven priorities [19,23]. Fifth, implementation science approaches should be used to translate policy intentions into actionable plans with clear indicators, budgets, timelines, and accountability mechanisms [26,30]. Sixth, transparency measures, conflict-of-interest policies, and public evidence hearings should be strengthened to safeguard policymaking from undue influence [19,27]. Finally, global frameworks such as WHO “Best Buys” and WHO-INTEGRATE should be adapted to Nepal’s context and accompanied by clear operational guidance for implementation [21,35].

Given Nepal’s ongoing federal transition, strengthening EIPM requires clearly defined and complementary roles across federal, provincial, and local governments. Federal institutions should provide strategic leadership through national evidence standards, research priority-setting, and integrated health information systems. Provincial governments should strengthen technical capacity, support cross-sectoral coordination, and adapt national policies to regional contexts, while local governments should be empowered to generate and use local data, engage communities in decision-making, and implement evidence-informed interventions tailored to local needs and socio-cultural realities. Strengthening accountability, communication, and coordination across all three levels of government is essential to address fragmentation, improve policy coherence, and ensure consistent use of evidence throughout the policy cycle.

Equity, contextualisation, and community engagement should remain central to these efforts. Evidence-aligned but context-insensitive policies may experience limited uptake and exacerbate inequities. Embedding equity assessments and meaningful stakeholder participation throughout agenda setting, policy formulation, implementation, and evaluation can strengthen legitimacy, ownership, and accountability. Similarly, investments in digital health architecture, including real-time reporting systems, decision-support tools, and community-based digital platforms, should be accompanied by workforce development and governance safeguards to ensure reliable and responsible use of evidence [19,32].

### Strengths and limitations

Strengths of this review include consolidation of multi-sectoral, multi-level evidence from policy environments, programs, and ethics, offering a holistic map of barriers and facilitators in Nepal. The structured synthesis across themes yields actionable categories for reform and investment. Limitations include heterogeneity of study designs (predominantly qualitative/descriptive), potential publication bias towards certain topics (e.g., NCDs, MNH), and variable timeframes that complicate contemporaneous interpretation [19,23,24]. Moreover, operational indicators of EIPM like timeliness, citation practices, implementation fidelity; were rarely reported, constraining quantitative synthesis [26]. Some included studies were multi-country analyses that included Nepal as one of the country cases. Therefore, certain barriers and facilitators may reflect broader LMIC experiences rather than challenges unique to Nepal, although findings were interpreted alongside Nepal-specific evidence.

### Recommendations for future research

Future research should focus on developing and evaluating approaches to assess evidence-informed policymaking across federal, provincial, and local levels in Nepal. Additional studies are needed to evaluate the effectiveness of evidence synthesis mechanisms, policy dialogues, and other knowledge translation strategies in supporting policy decisions. Comparative research exploring different approaches to evidence dissemination and stakeholder engagement may help identify contextually appropriate strategies for promoting evidence use in policymaking. Further research is also needed to examine governance, financing, and implementation challenges in priority policy areas, including non-communicable diseases, maternal and neonatal health, nutrition, and climate-health, within Nepal’s federal health system.

## Conclusion

In conclusion, Nepal’s EIPM landscape reflects strong aspiration but uneven institutionalization. Addressing the persistent barriers; such as political interference, fragmented governance, capacity constraints, and weak dissemination; requires governance clarity, capacity investments, and operational detail so that research does not stop at citation but translates into equitable, feasible, and accountable action. Institutionalizing EIPM ultimately entails building the systems that use evidence, not only producing more evidence.

## Supporting information

S1 File Additional file 1: PRISMA Checklist. (DOCX)

S2 File Additional file 2: Search strategy for each database. (PDF)

S1 Table Supplementary Table (1): Characteristics of included studies. (DOCX)
