# Supplementary material for: Barriers and facilitators of evidence-informed health policymaking in Nepal: A scoping review
Source: PLoS One. 2026 Oct 26;21(10):e0358730. doi: 10.1371/journal.pone.0358730 (PMC13704804; doi:10.1371/journal.pone.0358730)
Supplement: Supplementary file 1 [file pone.0358730.s002.pdf]

PubMed Hints **854**

((((("health policymaker*"[All Fields] OR "health administrator*"[All Fields] OR "health researcher*"[All Fields] OR "health research personnel"[All Fields] OR "health stakeholder*"[All Fields]) AND "barrier*"[All Fields]) OR "facilitator*"[All Fields] OR "challenge*"[All Fields] OR "opportunit*"[All Fields] OR "enabler*"[All Fields]) AND "evidence generation"[All Fields] OR "Evidence-Informed"[All Fields]) OR "knowledge generation"[All Fields] OR "knowledge translation"[All Fields] OR "evidence translation"[All Fields] OR "Engagement"[All Fields] OR "policy implementation"[All Fields] OR "policy formation"[All Fields] OR "policy making"[All Fields] OR "health systems"[All Fields] OR "health sector"[All Fields]) AND "nepal"[All Fields]) AND (2015:2026[pdat])

Embase Hints **674**

(((((("health policymaker*" or "health administrator*" or "health researcher*" or "health research personnel" or "health stakeholder*") and "barrier*") or "facilitator*" or "challenge*" or "opportunit*" or "enabler*") and "evidence generation") or "Evidence-Informed" or "knowledge generation" or "knowledge translation" or "evidence translation" or "Engagement" or "policy implementation" or "policy formation" or "policy making" or "health systems" or "health sector") and "nepal").mp. [mp=title, abstract, heading word, drug trade name, original title, device manufacturer, drug manufacturer, device trade name, keyword heading word, floating subheading word, candidate term word]

Scopus Hints- **127**

"health policymaker*" OR "health administrator*" OR "health researcher*" OR "health research personnel" OR "health stakeholder*" AND "barrier*" OR "facilitator*" OR "challenge*" OR "opportunit*" OR "enabler*" AND "evidence generation" OR "Evidence-Informed" OR "knowledge generation" OR "knowledge translation" OR "evidence translation" OR "Engagement" OR "policy implementation" OR "policy formation" OR "policy making" OR "health systems" OR "health sector" AND "nepal" AND (LIMIT-TO (DOCTYPE , "ar")) AND (LIMIT-TO (SUBJAREA , "MEDI"))

Global Health **484**

"health policymaker" OR "health administrator" OR "health researcher" OR "health research personnel" OR "health stakeholder" AND barrier* OR facilitator* OR challenge* OR opportunit* OR enabler* AND "evidence generation" OR "Evidence-

Informed" OR "knowledge generation" OR "knowledge translation" OR "evidence translation" OR "Engagement" OR "policy implementation" OR "policy formation" OR "policy making" OR "health systems" OR "health sector" AND "nepal"
