# Supplementary material for: Barriers and facilitators of evidence-informed health policymaking in Nepal: A scoping review
Source: PLoS One. 2026 Oct 26;21(10):e0358730. doi: 10.1371/journal.pone.0358730 (PMC13704804; doi:10.1371/journal.pone.0358730)
Supplement: Supplementary file 3 [file pone.0358730.s003.docx]

| **Item** | **Methods** | **Population** | **Results: Barriers** | **Results: Facilitators** | **Results: Other** |
| --- | --- | --- | --- | --- | --- |
| Paudel et al. (2019) (1)  **Study design** Policy documents review | **Time frame** 2000-2015  **Data collection** Policy documents  **Data analysis** qualitative content analysis | **Sample population** Six key policy documents related to maternal and neonatal survival  **Setting** Policy context in Nepal  **Discipline** Maternal and neonatal survival | **Barriers to use of evidence** Mismatch between values and strategy  Little attention to socio-cultural and geographic context  Limited stakeholder participation  Lack of integration and vertical siloing  Inadequate surveillance for equity | **Facilitators to use of evidence** International goal alignment  Use of national data  Adoption of international standards  Financial incentives | **Types of evidence used** National level policy documents   1) Integrated Management of Newborn and Childhood Illness; Programme Management Module (Training Package) 2) Maternal and Perinatal Death Surveillance and Response (MPDSR) Guideline 3) Mother’s Protection Program Implementation Guideline 4) National Safe Motherhood and Newborn Health Long Term Plan 5) National Policy on Skilled Birth Attendant 6) National Neonatal Health Strategy   **Types of result**  Proposed factors |
| Witter et al. (2019) (2)  **Study design** Case study  Qualitative study | **Time frame** 2017  **Data collection** Semi-structured interviews  Documentary analysis  **Data analysis** framework approach | **Sample population** Politicians  Technical staffs  Development partners  Non-government organizations  Academics and consultants  **Setting** Policy environment in eight low- and middle-income country including Nepal  **Discipline** Health systems | **Barriers to use of evidence** Fragmentation in the sector  Unclear role  Infrastructure gap  Conflicting external advice  Limited Operational Detail | **Facilitators to use of evidence** Proactive identification of evidence needs by the country  Facilitating learning across countries  Donor funding and endorsement  Not implying large structural changes | **Types of evidence used** Policies and programs  **Types of result** suggestions/proposed factors  Experiences |
| Dhimal et al. (2016) (3)  **Study design** Qualitative study | **Time frame** 2001-2007  **Data collection** Literature review Semi-structured interviews  **Data analysis** Thematic analysis | **Sample population** Health planners,  Policy makers  Researchers  **Setting** Policy environments in Nepal  **Discipline** Policy level interventions including women’s health, information, education and communication (IEC) programs and activities related to health service delivery issues, clinical/laboratory service issues, training and human resource development, health research programs | **Barriers to use of evidence** Communication gaps between researchers and policy makers.  Concerns over the quality, validity, and reliability of research conducted in Nepal.  Irrelevance of research findings to national policy needs (too micro-level or donor-driven).  Poor availability and documentation of research findings.  Time constraints faced by policy makers for accessing and reviewing lengthy reports.  Lack of specific and feasible recommendations in research reports | **Facilitators to use of evidence** Establishing a mechanism (like a Unit of Evidence Synthesis at NHRC) for synthesizing evidence and producing actionable messages.  Creating a supportive environment for evidence-based policy, including giving priority to health research.  Developing methods for easy access, such as a digital library.  Promoting regular interaction and discussion between researchers and policymakers.  Implementing quality assurance practices, such as a Monitoring and Evaluation section in NHRC, to ensure reliability. | **Types of evidence used** Policies and programs  **Types of result** suggestions/proposed factors  Perceptions |
| Lecky et al. (2020) (4)  **Study design** Research prioritisation setting | **Time frame** 2019  **Data collection**  Focus Group  **Data analysis** Modified nominal group  techniques | **Sample population** Researchers  Stakeholders  Representatives from Ministry of Health  **Setting** Policy environment low- and middle-income countries including Nepal  **Discipline** Emergency care | **Barriers to use of evidence** External environment  Regulation, policy, local settings, bureaucracy  Research community  Brain drain, access to papers, time, collaboration, research capacity  Conduct of research  Data collection, data quality, research implementation | **Facilitators to use of evidence**  Political mandate and need for evidence  Collaborative networks  Inclusivity and contextual leadership  Structured prioritisation | **Types of evidence used** Consensus and expert opinion  **Types of result** Perceptions |
| Hipgrave et al.  (2019) (5)  **Study design** Mixed methods | **Time frame** 2014  **Data collection** Literature review Semi-structured interviews  Government spending data  **Data analysis** Political economy analysis  Budget analysis | **Sample population** Government officials/ leaders/experts in health and other social sectors, finance and planning  **Setting** Policy environment in Bangladesh, Indonesia, Nepal and the Philippines  **Discipline** Maternal, newborn and child health | **Barriers to use of evidence** Political commitment/priority  Poor governance and decentralization  Evidence and resource allocation  Weak public sector human resource capacity | **Facilitators to use of evidence**  Consistent donor funding  Political commitment  Well-informed media coverage  Private sector regulation and engagement | **Type of evidence used**  Primary research |
|  |  |  |  |  | **Types of result** Perceptions/experiences |
| Pradhan PMS et.al. (2021) (6)  **Study design** Qualitative | **Time frame** 2018  **Data collection** desk review  Key informant interviews  **Data analysis** SWOT | **Sample population** Government officials  **Setting** Policy environment in Nepal  **Discipline** Cardiovascular health | **Barriers to use of evidence** Lack of multi-sectoral coordination  Scarcity of cardiovascular disease-specific protocols. | **Facilitators to use of evidence** Voice and accountability from local level  Government willingness and strategy to work together with the private and non-government sectors  Regulatory quality  Control of corruption  Financial transparency | **Types of evidence used** Guidelines, law and policies  **Types of result** Perception/experiences |
| Khanal et al. (2025) (7)  **Study design** Qualitative | **Time frame** 2023  **Data collection** semi-structured interviews  **Data analysis** Thematic analysis | **Sample population** Government officials  **Setting** Policy environment in Nepal  **Discipline** Health and climate change | **Barriers to use of evidence** Knowledge gaps  Ownership and accountability  Resource constraints: human resource and budget  Data limitations and their use for decision making | **Facilitators to use of evidence** Acknowledgement of the need to integrate health equity and climate change  Role of political leadership  Global influences  Established mechanisms and structures for collaboration in place  The federal structure | **Types of evidence used** Health and climate change policies  **Types of result** Perceptions /experiences |
| Buse et al. (2020) (8)  **Study design** Qualitative study | **Time frame** 2000-2019  **Data collection** Literature review  Key informant interview  **Data analysis** Policy content analysis | **Sample population** Government officials  **Setting** Policy environment in Afghanistan, Bangladesh, Nepal, Pakistan, Tunisia and Vietnam  **Discipline** Non-communicable diseases | **Barriers to use of evidence**  Weak accountability  Limited budgets  Fragmented coordination  Low policy authority | **Facilitators to use of evidence** Multi-sectoral NCD committees  WHO technical support  Existing strategic frameworks. | **Types of evidence used** World Health Organization ‘Best Buys’ guidelines for the prevention and control of noncommunicable diseases (NCDs)  **Types of result** Documentary proof of evidence use |
| Tiwari et al. (2021) (9)  **Study design** Qualitative study | **Time frame** 2018  **Data collection**  Semi-structured interviews  **Data analysis** Thematic analysis | **Sample population** Policy-makers  Health researchers  **Setting** Policy environment in Nepal  **Discipline** Health policy | **Barriers to use of evidence** Political influence  Poor culture of evidence utilization  Preference of anecdotal evidences  Donor agency’s influence  Poor expenditure for research  Issues of generalizability and credibility of evidences  Poor quality and trust of evidences  Low availability of priority-based researches Poor targeted dissemination | **Facilitators to use of evidence** Targets and commitments made at international conferences  Expert/ Stakeholder’s consultation  Availability of nationally representative surveys  Publication of one-pager research  Partial cost effectiveness study | **Types of evidence used** National policy agenda  **Types of result** Perceptions |
| Namirembe et al (2022) (10)  **Study design** Quantitative study | **Time frame** 2016  **Data collection** Survey  **Data analysis** Principal component analysis | **Sample population** Government and non-government officials  **Setting** Policy environment in Nepal  **Discipline** Nutrition | **Barriers to use of evidence** Weak intersectoral coordination  Resource disparities  Limited ownership | **Facilitators to use of evidence** Strong political commitment  Multisector Nutrition Plan (MSNP)  Local leadership and capacity building | **Types of evidence used** World Health Organization’s Nutrition Governance  Hunger and Nutrition Commitment Index (HANCI)  Political Commitment (for Nutrition) Rapid Assessment Tool.  **Types of result** Perceptions |
| Sapkota et al. (2022) (11)  **Study design** Qualitative | **Time frame** 2020  **Data collection**  Policy document review  Focus group discussions  In-depth interviews  **Data analysis** Content analysis | **Sample population** Health workers  Policy makers  Program managers  Service providers  **Setting** Policy environment in Nepal  **Discipline** Non-communicable diseases | **Barriers to use of evidence** Fragmented governance  Workforce shortages.  Poor logistics and financing | **Facilitators to use of evidence** PEN program rollout  National policies  Political commitments to non-communicable diseases interventions | **Types of evidence used** Multi-sectoral Action Plan (2014–2020).  PEN implementation and NCD surveillance  **Types of result** Perceptions/experiences |
| Bhattarai et al. (2022) (12)  **Study design** Mixed methods | **Time frame** 2021  **Data collection** Survey  Key informant interviews  Focus group discussions  **Data analysis** Delphi | **Sample population** Health workforce  Government and non-government officials  **Setting** Clinical setting in Nepal  **Discipline** COVID-19, clinical ethics | **Barriers to use of evidence** Political and bureaucratic interference | **Facilitators to use of evidence** Rational allocation of medical resource, Updated treatment protocols that guide clinical decisions  Standard-of-care regardless of patient’s economic status  Effective communication among stakeholders for prompt patient care | **Types of evidence used** Guidelines |
|  |  |  |  |  | **Type of result**  Perceptions/experiences |
| Gaihre et al. (2019) (13)  **Study design** Qualitative | **Time frame** 2015  **Data collection** Participatory workshop  **Data analysis** Thematic analysis | **Sample population** Researchers  Government and non-government officials  Civil society  **Setting** Policy environment in Nepal  **Discipline** Food security  Nutrition  Population health | **Barriers to use of evidence**  Fragmentation  Lack of research-based and practice-based evidence  Limited transdisciplinary knowledge amongst sectoral stakeholders  Short-term funding and lack of knowledge-sharing mechanisms | **Facilitators to use of evidence**  Improved knowledge amongst policymakers and implementers  Investment in collaborative practice-based research and evidence-based practice Strengthened transdisciplinary collaboration between stakeholders | **Types of evidence used** Multisector Nutrition Plan (MSNP) ‘Sunaula Hazar Din’ Golden Thousand Days (GTD): Community Action for Nutrition  **Type of results**  Perceptions/experiences |
| Stratil et al (2020) (14)  **Study design** Qualitative study | **Time frame** 2017  **Data collection** Key informant interviews  Focus group discussions  **Data analysis** Content analysis | **Sample population** Government and non-government officials  **Setting** Policy environment in Brazil, Germany, Nepal and Uganda  **Discipline** Sexual and reproductive health; health services research | **Barriers to use of evidence** Socio-cultural acceptability  Capacity of healthcare provider | **Facilitators to use of evidence** Local coordination  User friendliness | **Types of evidence used** WHO-INTEGRATE Framework  **Types of result** Perceptions/experiences |
| Archana et al. (2019) (15)  **Study design** Qualitative study | **Time frame** Not stated  **Data collection** Focus group discussions  Workshops  **Data analysis** Thematic | **Sample population** Patients and the Public, Providers, Purchasers, Payers, Public Policy Makers and Policy Advocates, Product Makers and the Principal Investigators  **Setting** Policy environment in Nepal  **Discipline** Cardiovascular disease | **Barriers to use of evidence** Political uncertainty  Conflicting priorities of stakeholders  Committing time for engagement | **Facilitators to use of evidence**  Continuous stakeholder engagement  Provide clear information to stakeholders  Clear role and responsibilities  Dedicated time | **Types of evidence used** WHO Package of Essential Noncommunicable Diseases Interventions (WHO PEN)  **Types of result** Perceptions/Experiences |
| Fisher et al. (2021)  (16)  **Study design** Qualitative | **Time frame** 2018  **Data collection** Policy documents review  Key informant interviews  **Data analysis** Content analysis | **Sample population** Government and non-government officials  Medical Professionals  Researchers  Media  **Setting** Policy environment in Nepal  **Discipline** public health | **Barriers to use of evidence** Private sector interference in policymaking  Lack of international actor support  Absence of well-designed and enacted policies and legislation  Lack of political commitment to regulate Insufficient mobilisation of existing evidence to spur action  Lack of national evidence to guide regulatory design  Weak implementation capacity | **Facilitators to use of evidence** Ability to combat private sector interference | **Types of evidence used** WHO Marketing Recommendations and the ‘best buys’ interventions  **Types of result** Perceptions/Experiences |
| Dhimal et al.  (2016) (17)  **Study design** Qualitative | **Time frame** 2016  **Data collection** Key informant interview  **Data analysis** Thematic analysis | **Sample population** Researchers  Policy-makers  **Setting** Policy environment in Nepal  **Discipline** social care / social work | **Barriers to use of evidence** Timing/opportunity  Policy-maker research skills | **Facilitators to use of evidence** contact with researchers/info staff  Contact with policy makers  relationship with researchers/info staff  Relationship with policy-makers  Collaboration  Managerial support (practical)  Other | **Types of evidence used** Primary research  **Types of result** Perceptions |
| Tiwari et al (2023) (18)  **Study design** Mixed methods | **Time frame** 2014-2018  **Data collection** Surveys  Semi-structured interviews  **Data analysis** Poisson regression  RE-AIM | **Sample population** Reproductive-aged women  Community health nurses  Community health program associates  Program staffs    **Setting** Community healthcare in Nepal  **Discipline** Maternal healthcare | **Barriers to use of evidence**  Lack of trust  Timely care | **Facilitators to use of evidence** Full-time, supervised, and trained community health workers  Mobile tools | **Types of evidence used** Primary research  **Types of result** Experiences |
| Khatri et al. (2023) (19)  **Study design** Qualitative | **Time frame** 2018-2019  **Data collection** Key informant interview  **Data analysis** Braun and Clarke’s thematic approach | **Sample population** Health policy makers Program managers  **Setting** Policy environment in Nepal  **Discipline** Maternal and newborn health services | **Barriers to use of evidence**  Corruption and poor accountability  Weak digital governance and institutionalisation of policies  Politicisation of the health workforce  Poor regulation of private maternal and new born health (MNH) services  Weak health management  Lack of integration of health in all policies  Weak decentralisation  Inadequate evidence-based planning  Lack of contextualizing health services  Poor quality health care  Inadequate empowerment in household decision making  Lack of community participation. | **Facilitators to use of evidence** Local recruitment of health workforce  Inclusion in local health committees  Decentralisation and institutional reform Evidence based contextual planning  Use of digital tools and health information systems  Intersectoral collaboration,  Strengthening local management capacity Community empowerment and ownership. | **Types of evidence used**  National policies  **Types of result** Experiences/Perceptions |
| Binagwaho et al. (2024)  (20)  **Study design** Case study | **Time frame**  2017-2020  **Data collection** Desk review  Key informant interview  **Data analysis** Content analysis | **Sample population** Government and non-government officials  **Setting** Policy environment in six low- and middle-income countries including Nepal  **Discipline** Under-5 mortality | **Barriers to use of evidence** Geography  Health insurance  Culture and beliefs  Health systems structure and strength  Financial commitment to the health sector | **Facilitators to use of evidence** Donor funding priorities and availability  Global implementation tools (such as guidelines)  Culture of donor and partner coordination  Economic growth  Financial commitment to the health sector  Health systems structure and strength  In-country research capacity  Leadership and governance and a culture of accountability  National priority for health and primary care Non-health national infrastructure and systems strengthening  Preexisting culture and capacity of data use Strong preexisting community health system and structure including CHWs | **Types of evidence used** Literature and guidelines on evidence-based interventions  **Types of result** Experiences/Perceptions |
| Wasti et al.  (2023) (21)  **Study design**  Qualitative | **Time frame**  2021 | **Sample population**  Policymakers, health service providers, local elected members, and other local stakeholders. | **Barriers to use of evidence**  Poor coordination between the tiers of government  Delayed release of funds  Maldistribution of staff  Problems over procurement  Limited monitoring and supervision of the quality-of-service delivery  Data reporting | **Facilitators to use of evidence**  Tailored local health policies and plans Improved health governance at the municipality level  Improved health infrastructure and service capacity  improved outreach services  Increased resources  Improved real-time data reporting from health facilities | **Type of evidence used**  National policy  **Type of result**  Experiences/Perceptions |
|  | **Data collection**  Key informant interviews Participatory policy analysis workshops | **Setting**  Policy environment in Nepal |  |  |  |
|  | **Data analysis**  Thematic analysis using WHO health systems framework | **Discipline**  Health system |  |  |  |
| Shrestha et al.  2025 (22)  **Study design**  Qualitative | **Time frame**  2024 | **Sample population**  Politician, regulatory  bodies, government officials, academy, professional societies | **Barriers to use of evidence**  Political and Bureaucratic Challenges  Financial hurdles | **Facilitators to use of evidence**  Collaboration and Stakeholder Engagement  Commitment, Accountability, and Leadership  Follow-up Mechanism | **Type of evidence used**  **Nation health policy**  **Type of result**  Experiences/Perceptions |
|  | **Data collection**  Key informant interviews | **Setting**  Policy environment in Nepal |  |  |  |
|  | **Data analysis**  Thematic analysis | **Discipline**  Health system |  |  |  |
| Pomeroy-Stevens et al.  2016 (23)  **Study design**  Mixed method | **Time frame**  2015-2016  **Data collection**  Key informant interviews  Tracking budget data  **Data analysis**  Track qualitative and budgetary  changes related to Nepal’s multisectoral nutrition plan (MSNP) processes | **Sample population**  Government, donors, UN groups, civil society organizations (CSOs), academia, and the private sector | **Barriers to use of evidence**  Human resources  Bottom-up planning  Lack of ownership | **Facilitators to use of evidence**  Multisectoral coordination of nutrition activities  High-level advocacy for nutrition  Sustainable structures | **Type of evidence used**  Nepal’s multisectoral nutrition plan  **Type of result**  Perceptions/Knowledge/Attitudes |
|  |  | **Setting**  Policy environment in Nepal |  |  |  |
|  |  | **Discipline**  Nutrition |  |  |  |
| Sato et al.  2015 (24)  **Study design**  Qualitative | **Time frame**  2008-2009  **Data collection**  Document review  Interviews  **Data analysis**  Content analysis | **Sample population**  Managers at district offices Facility managers or senior-level staff  Health workers  Members of the Health Management Committees (HMCs) who had been involved in the free health-care policy (FHCP) implementation  **Setting**  Policy environment in Nepal  **Discipline**  Health system | **Barriers to use of evidence**  Insufficient or delayed inputs of drugs and compensation  Insufficient workforce  Reduced quality of services | **Facilitators to use of evidence**  Good intra- and inter-facility relationships  Adequate staffing  Well-oriented providers  Skilled health management committees | **Type of evidence used**  Free health-care policy (FHCP)  **Type of result**  Experiences/Perceptions |
| M. Lamichhane Dhimal et al.  2025 (25)  **Study design**  Qualitative | **Time frame**  2023  **Data collection**  Document review  Interviews  **Data analysis**  Thematic analysis | **Sample population**  Government policymakers, private sector and civil society  **Setting**  Policy environment  **Discipline**  Health system and climate change in Nepal | **Barriers to use of evidence**  Poor data, weak intersectoral collaboration, insufficient capacity and inadequate funding | **Facilitators to use of evidence**  Strengthen inter-ministerial and cross-disciplinary collaboration | **Type of evidence used**  Grey and peer- reviewed literature, policy document related to climate change and health research  **Type of result**  Experiences/Perceptions |
| Rawal LB, et al.  2020 (26)  **Study design**  Qualitative | **Time frame**  2017  **Data collection**  Document review  Interviews  Consultation meetings  **Data analysis**  Thematic analysis | **Sample population**  Policy makers  Community health workers  Staffs from government and non-government organizations  Private sector  Academia  **Setting**  Policy environment in Nepal  **Discipline**  Non-communicable diseases (NCDs) | **Barriers to use of evidence**  Inadequate training, high workload, poor system-level support, inadequate remuneration, inadequate supply of logistics and drugs | **Facilitators to use of evidence**  Government priority, formation of NCD-related policies, community support systems, social prestige and staff motivation | **Type of evidence used**  WHO Package for Essential Non communicable disease prevention and control (PEN) programs  **Type of result**  Experiences/Perceptions |

**References**

1) Paudel, M., Javanparast, S., Dasvarma, G. *et al.* A critical account of the policy context shaping perinatal survival in Nepal: policy tension of socio-cultural versus a medical approach. *BMC Health Serv Res* **19**, 166 (2019). <https://doi.org/10.1186/s12913-019-3979-3>
2) Witter, S., Anderson, I., Annear, P. *et al.* What, why and how do health systems learn from one another? Insights from eight low- and middle-income country case studies. *Health Res Policy Sys* **17**, 9 (2019). <https://doi.org/10.1186/s12961-018-0410-1>
3) Dhimal, M., Pandey, A. R., Aryal, K. K., Budhathoki, C. B., Vaidya, D. L., Karki, K. K., & Onta, S. (2016). Utilization of Health Research Recommendation in Policy and Planning in Nepal. *Journal of Nepal Health Research Council*, *14*(34), 165–172.
4) Lecky, F.E., Reynolds, T., Otesile, O. *et al.* Harnessing inter-disciplinary collaboration to improve emergency care in low- and middle-income countries (LMICs): results of research prioritisation setting exercise. *BMC Emerg Med* **20**, 68 (2020). <https://doi.org/10.1186/s12873-020-00362-7>
5) Hipgrave, D. B., Anderson, I., & Sato, M. (2019). A rapid assessment of the political economy of health at district level, with a focus on maternal, newborn and child health, in Bangladesh, Indonesia, Nepal and the Philippines. *Health policy and planning*, *34*(10), 762–772. <https://doi.org/10.1093/heapol/czz082>
6) Pradhan, P. M., , Bhatt, R. D., , Pandit, R., , Shrestha, A., , Maharjan, R., , Jha, N., , Bajracharya, S., , Shrestha, S., , Koju, R. P., , & Karmacharya, B. M., (2021). Needs Assessment of Leadership and Governance in Cardiovascular Health in Nepal. *Kathmandu University medical journal (KUMJ)*, *19*(75), 30–39.
7) Khanal, S., Baral, S.C. & Boeckmann, M. Exploring barriers and facilitators to integrating health equity into health and climate change policies in Nepal – a qualitative study among federal level stakeholders. *BMC Health Serv Res* **25**, 687 (2025). <https://doi.org/10.1186/s12913-025-12862-y>
8) Buse, K., Aftab, W., Akhter, S., Phuong, L. B., Chemli, H., Dahal, M., Feroz, A., Hofiani, S., Pradhan, N. A., Anwar, I., Skhiri, H. A., El Ati, J., Giang, K. B., Puri, M., Noormal, B., Rabbani, F., & Hawkes, S. (2020). The state of diet-related NCD policies in Afghanistan, Bangladesh, Nepal, Pakistan, Tunisia and Vietnam: a comparative assessment that introduces a 'policy cube' approach. *Health policy and planning*, *35*(5), 503–521. <https://doi.org/10.1093/heapol/czz175>
9) Tiwari, B.B., Ban, A., Gurung, S. *et al.* Translating evidence into policy: opinions and insights of Health Researchers and Policymakers in Nepal. *BMC Health Serv Res* **21**, 1066 (2021). <https://doi.org/10.1186/s12913-021-07102-y>
10) Namirembe, G., Shrestha, R., Webb, P., Houser, R., Davis, D., Baral, K., Mezzano, J., & Ghosh, S. (2022). Measuring Governance: Developing a Novel Metric for Assessing Whether Policy Environments are Conducive for the Development and Implementation of Nutrition Interventions in Nepal. *International journal of health policy and management*, *11*(3), 362–373. <https://doi.org/10.34172/ijhpm.2020.135>
11) Sapkota BP, Baral KP, Berger U, Parhofer KG, Rehfuess EA (2022) Health sector readiness for the prevention and control of noncommunicable diseases: A multi-method qualitative assessment in Nepal. PLoS ONE 17(9): e0272361. [https://doi.org/10.1371/journal. pone.0272361](https://doi.org/10.1371/journal.%20pone.0272361)
12) Bhattarai S, Adhikari A, Rayamajhee B, Dhungana J, Singh M, Koirala S and Shakya DR (2022) Participatory Approach to Develop Evidence-Based Clinical Ethics Guidelines for the Care of COVID-19 Patients: A Mixed Method Study From Nepal. Front. Public Health 10:873881. doi: 10.3389/fpubh.2022.873881
13) Gaihre, S., Kyle, J., Semple, S. *et al.* Bridging barriers to advance multisector approaches to improve food security, nutrition and population health in Nepal: transdisciplinary perspectives. *BMC Public Health* **19**, 961 (2019). <https://doi.org/10.1186/s12889-019-7204-4>
14) Stratil, J. M., Paudel, D., Setty, K. E., Menezes de Rezende, C. E., Monroe, A. A., Osuret, J., Scheel, I. B., Wildner, M., & Rehfuess, E. A. (2022). Advancing the WHO-INTEGRATE Framework as a Tool for Evidence-Informed, Deliberative Decision-Making Processes: Exploring the Views of Developers and Users of WHO Guidelines. *International journal of health policy and management*, *11*(5), 629–641. <https://doi.org/10.34172/ijhpm.2020.193>
15) Archana, S., Karmacharya, B. M., Rashmi, M., Abhinav, V., Meghnath, D., Natalia, O., Rajeev, S., Prajjwal, P., Annette, F., David, C., Swornim, B., Roman, X. D., Donna, S., & Rajendra, K. (2019). Stakeholder Engagement in Planning the Design of a National Needs Assessment for Cardiovascular Disease Prevention and Management in Nepal. *Global heart*, *14*(2), 181–189. <https://doi.org/10.1016/j.gheart.2019.05.002>
16) Fisher, L., Dahal, M., Hawkes, S. *et al.* Barriers and opportunities to restricting marketing of unhealthy foods and beverages to children in Nepal: a policy analysis. *BMC Public Health* **21**, 1351 (2021). <https://doi.org/10.1186/s12889-021-11257-y>
17) Dhimal M, Pandey AR, Aryal KK, Subedi M, Karki KB. Translation of Health Research Evidence into Policy and Planning in Nepal: An Appraisal 2016. Kathmandu, Nepal: Nepal Health Research Council, 2016.
18) Tiwari A, Thapa A, Choudhury N, Khatri R, Sapkota S, Wu W-J, et al. (2023) A Type II hybrid effectiveness-implementation study of an integrated CHW intervention to address maternal healthcare in rural Nepal. PLOS Glob Public Health 3(1): e0001512. [https://doi.org/10.1371/journal. pgph.0001512](https://doi.org/10.1371/journal.%20pgph.0001512)
19) Khatri, R.B., Assefa, Y. & Durham, J. Multidomain and multilevel strategies to improve equity in maternal and newborn health services in Nepal: perspectives of health managers and policymakers. *Int J Equity Health* **22**, 105 (2023). <https://doi.org/10.1186/s12939-023-01905-7>
20) Binagwaho, A., VanderZanden, A., Garcia, P.J. *et al.* Cross-country analysis of contextual factors and implementation strategies in under-5 mortality reduction in six low- and middle-income countries 2000–2015. *BMC Pediatr* **23** (Suppl 1), 652 (2024). <https://doi.org/10.1186/s12887-023-03906-5>
21) Wasti, S. P., van Teijlingen, E., Rushton, S., Subedi, M., Simkhada, P., Balen, J., & Nepal Federal Health System Team (2023). Overcoming the challenges facing Nepal's health system during federalisation: an analysis of health system building blocks. *Health research policy and systems*, *21*(1), 117. <https://doi.org/10.1186/s12961-023-01033-2>
22) Shrestha, A., Poudel, L., Tikhatri, D., Regmi, S., Jha, J. B., Baniya, S., Phuyal, M. P., Prasai, A., Kunwar, S., Chand, A., Karki, P., Tamrakar, N., Sapkota, R., & Yadav, R. (2025). Tracking Progress of National Health Summit Declarations - Health Policy Analysis: A Qualitative Study. *JNMA; journal of the Nepal Medical Association*, *63*(281), 3–11. <https://doi.org/10.31729/jnma.8871>
23) Pomeroy-Stevens, A., Shrestha, M. B., Biradavolu, M., Hachhethu, K., Houston, R., Sharma, I., & Wun, J. (2016). Prioritizing and Funding Nepal's Multisector Nutrition Plan. *Food and nutrition bulletin*, *37*(4 suppl), S151–S169. <https://doi.org/10.1177/0379572116674555>
24) Sato, M., & Gilson, L. (2015). Exploring health facilities' experiences in implementing the free health-care policy (FHCP) in Nepal: how did organizational factors influence the implementation of the user-fee abolition policy?. *Health policy and planning*, *30*(10), 1272–1288. <https://doi.org/10.1093/heapol/czu136>
25) Lamichhane Dhimal, M., Bhandari, D., Ghimire, S., Dahal Khatri, B., Shrestha, R., Sapkota, A., Ebi, K. L., Marten, R., & Dhimal, M. (2025). Building climate-resilient and low-carbon health systems in nepal: Insights from a policy document review and qualitative interviews with key stakeholders. *Public Health, 249*, 106001. [https://10.1016/j.puhe.2025.106001](https://10.0.3.248/j.puhe.2025.106001)
26) Rawal LB, Kharel C, Yadav UN, et al Community health workers for non-communicable disease prevention and control in Nepal: a qualitative studyBMJ Open 2020;10:e040350. doi: 10.1136/bmjopen-2020-040350
