# Supplementary material for: Barriers and facilitators of evidence-informed health policymaking in Nepal: A scoping review
Source: PLoS One. 2026 Oct 26;21(10):e0358730. doi: 10.1371/journal.pone.0358730 (PMC13704804; doi:10.1371/journal.pone.0358730)
Supplement: Supplementary file 4 [file pone.0358730.s005.docx]

Editorial Team

PLOS One

13^th^ August 2026

**Re: Manuscript reference number-PONE-D-26-00588**

Dear Editorial Team,

I am writing to submit a revision of the above manuscript. We sincerely appreciate the helpful comments from the reviewer, and we have revised the manuscript according to his/her suggestions.

The modified sections in the manuscript are highlighted using tracked changes. Please find the attached response to the reviewer which details the changes we have made along with explanations for these changes.

We hope that the revised manuscript will now be acceptable for publication in *PLOS One* and look forward to your decision. Thank you very much for your consideration of this manuscript.

Yours sincerely,

Dr Kaung Suu Lwin, MBBS MHSc PhD

School of Human and Health Sciences

University of Huddersfield | Queensgate | Huddersfield | HD1 3DH

**RESPONSE TO REVIEWERS**

We would like to thank the editor and reviewer for providing helpful comments. Our responses are given beneath each comment. The added/revised text is double-quoted in our response for ease of reference, with line and page numbers provided where necessary.

***Reviewer #1:***

1) *The search strategy is reported inconsistently. The abstract states that searches were conducted in PubMed, Scopus, Embase, and Global Health, whereas the Methods section lists PubMed, Scopus, Web of Science Core Collection, and NepJOL. The authors should clarify which databases were actually searched.*

Thank you for this observation. We apologize for the inconsistency. Searches were performed in PubMed, Scopus, Embase, and Global Health. The mention of Web of Science Core Collection and NepJOL in the Methods section was incorrect and has now been revised for consistency with the actual search strategy described in the abstract. The Methods section has been updated accordingly to accurately reflect the databases searched.

**Revised manuscript text (Page 4, Lines 119-124):**

**“Information Sources and Search Strategy**

An electronic search was conducted in October 2025 through PubMed, Scopus, Embase, and Global Health using the keywords: evidence-informed, knowledge translation, policy making, barriers, challenges, facilitators, opportunities and Nepal. These keywords were searched in Medline using the Boolean operators “AND” and “OR” (a detailed search strategy of each database is provided in additional file 1).”

2) *The PRISMA-ScR reporting should be strengthened. The manuscript reports that 2,139 records were identified, 48 full-text articles were screened, and 26 studies were included, but the screening and exclusion process is not sufficiently detailed. The authors should provide clearer information on duplicate removal, full-text exclusion reasons, and the study selection process.*

Thank you for this suggestion. We agree that the study selection process should be reported more transparently in accordance with the PRISMA-ScR guidelines. We have revised the manuscript to provide additional details on the screening process, including the number of duplicate records removed, the stages of title/abstract and full-text screening, and the reasons for full-text exclusions in the results. We have also expanded the description of the study selection procedure in the Methods section.

**Revised manuscript text in the Methods (Page 4, Lines 125-133):**

“**Study Selection**

All retrieved records were imported into Rayyan for reference management and duplicate removal. Following deduplication, two reviewers independently screened titles and abstracts against the predefined eligibility criteria. Records considered potentially relevant proceeded to full-text review. Full-text articles were independently assessed by the reviewers, and any disagreements were resolved through discussion and, where necessary, consultation with a third reviewer. Reasons for exclusion at the full-text screening stage were documented. The study selection process was conducted in accordance with PRISMA-ScR guidance and is illustrated in the PRISMA flow diagram (Figure 1).”

**Revised manuscript text in the Results (Page 5, Lines 156-162):**

**“Results**

A total of 2,139 records were identified through database searches. After the removal of 724 duplicate records, 1,415 records were screened by title and abstract. Following screening, 48 articles were retrieved and assessed for full-text eligibility. Of these, 22 articles were excluded for the following reasons: review articles without evidence used (n=6), protocol papers (n=2), synopsis article (n=1), absence of barriers or facilitators relevant to the review objective (n=4), no discussion of evidence-informed policies (n=4), and studies not conducted in Nepal (n=5). A total of 26 studies met the inclusion criteria and were included in the review. The complete study selection process is summarised in Figure 1.”

3) *The manuscript states that 26 studies were included, but the numbers reported by study design add up to 30: qualitative studies (n=18), mixed-methods studies (n=5), policy document reviews (n=4), and quantitative surveys (n=3). The authors should clarify whether these categories are mutually exclusive.*

Thank you for identifying this discrepancy. The study design categories were not intended to be mutually exclusive. Some included studies employed multiple methodologies and were therefore classified under more than one category. This resulted in the sum of study design classifications exceeding the total number of included studies (n = 26). To improve clarity, we have revised the manuscript to explicitly state that the categories overlap and that some studies contributed to more than one methodological category.

**Revised manuscript text (Page 6, Lines 169-172):**

“Study designs were predominantly qualitative (n = 18), complemented by mixed-methods studies (n = 5), policy document reviews (n = 4), and quantitative surveys (n = 3). As some studies were classified into more than one study design category, the totals exceed the 26 studies included in the review.”

4) *The thematic synthesis needs to be described more transparently. The manuscript states that text segments related to barriers and facilitators were coded and grouped into broader categories and overarching themes. However, it does not provide enough detail on what coding framework was used, how inductive coding was conducted, how disagreements between reviewers were resolved, and how the final themes were derived.*

Thank you for this comment. We have revised the Data Analysis section to provide greater detail on the thematic synthesis process. Specifically, we clarified that an inductive approach was used without a predefined coding framework, that two reviewers independently conducted line-by-line coding of extracted text segments, and that disagreements were resolved through discussion and consensus, with referral to a third reviewer where necessary. We also expanded the description of how codes were grouped into categories and iteratively refined into overarching themes.

**Revised manuscript text (Page 5, Lines 147-156):**

“For qualitative data, we conducted an inductive thematic synthesis. No predefined coding framework was applied; rather, codes were generated directly from the extracted data. Text segments related to barriers and facilitators were coded independently by two reviewers using a line-by-line approach. The reviewers compared coding outputs, and any discrepancies were resolved through discussion and consensus, with consultation from a third reviewer where necessary. Similar codes were subsequently grouped into descriptive categories, which were iteratively reviewed and refined to develop overarching themes reflecting systemic, institutional, and contextual factors influencing evidence-informed health policymaking in Nepal. The thematic synthesis followed an iterative process to ensure consistency, capture emerging patterns.”

5) *The authors should further develop the sector-specific interpretation of the findings. For example, it would be useful to explain whether certain barriers, such as donor-driven agendas, weak governance, or local socio-cultural norms, are more pronounced in specific policy areas such as non-communicable diseases, maternal and neonatal health, nutrition, or climate-health policy*

Thank you for this valuable suggestion. We agree that greater attention to sector-specific patterns strengthen the interpretation of the findings. We have revised both the Results and Discussion sections to highlight differences across policy domains. Specifically, we describe how socio-cultural and contextual barriers were more prominent in maternal and neonatal health, governance and coordination challenges were particularly evident in non-communicable disease and health systems policy, multisectoral coordination issues featured strongly in nutrition policy, and data, capacity, and funding constraints were especially salient in climate-health policy. We further discuss the implications of these sector-specific patterns for strengthening evidence-informed policymaking in Nepal.

**Revised manuscript text in the Results (Page 9, Lines 286-308):**

**“Policy Area-Specific Patterns in Barriers and Facilitators**

While several barriers and facilitators were observed across policy areas, some were more pronounced within specific health policy domains. In maternal and neonatal health, socio-cultural norms, limited community participation, weak decentralisation, and challenges in contextualising interventions were frequently identified as barriers to evidence-informed [16-20]. Key facilitators included community empowerment, local health committees, decentralised decision-making, and evidence-based contextual planning [16-20].

In non-communicable disease (NCD) policy, governance and implementation challenges were particularly prominent. Fragmented governance structures, weak accountability mechanisms, workforce shortages, financing constraints, and inadequate logistics were commonly reported, while political commitment, multisectoral coordination mechanisms, implementation of the WHO Package of Essential Noncommunicable Disease Interventions (PEN), and technical support from international partners facilitated evidence use in policymaking [21-25].

Nutrition-related policy studies highlighted weak multisectoral coordination, limited stakeholder ownership, resource disparities, and short-term funding arrangements as key barriers [26-28]. Facilitators included high-level advocacy, multisectoral nutrition platforms, local leadership, and capacity-building initiatives that supported cross-sectoral action [26-28].

In climate-health policy, knowledge gaps, limited availability and use of local data, insufficient technical capacity, inadequate funding, and weak intersectoral collaboration emerged as major challenges. Facilitators included political leadership, federal governance structures, cross-sectoral partnerships, and support from global actors and international policy frameworks [37,38].”

**Revised manuscript text in the Discussion (Page 11, Lines 356-366):**

An important finding of this review is that barriers and facilitators varied across policy domains. In maternal and neonatal health, evidence use was particularly influenced by socio-cultural norms, community participation, and the need for context-specific policy implementation [16-20]. In contrast, studies on non-communicable diseases primarily highlighted governance, financing, workforce, and coordination challenges, reflecting the multisectoral nature of NCD prevention and control [21-25]. Nutrition and climate-health policies relied heavily on multisectoral collaboration and external support, while climate-health studies additionally emphasized technical capacity, data limitations, and evidence gaps [26-28,37,38]. These findings suggest that although strengthening governance, evidence generation, and knowledge translation is important across all policy areas, tailored approaches are needed to address sector-specific challenges and opportunities.

6) *The policy implications should be made more specific. Many of the identified barriers and facilitators are common across low- and middle-income countries. The manuscript would be stronger if it explained more clearly how Nepal’s federalized health governance system shapes these barriers and what roles should be played by national, provincial, and local institutions.*

We thank the reviewer for this comment. We agree that the policy implications should more clearly reflect Nepal's federalised health governance structure. We have therefore revised the Policy Implications section to specify the complementary roles of federal, provincial, and local institutions in strengthening evidence-informed policymaking. The revised text highlights the federal role in setting standards and coordinating evidence systems, the provincial role in policy adaptation and technical support, and the local role in generating and applying context-specific evidence. We also emphasise the importance of intergovernmental coordination and accountability mechanisms to address the fragmentation identified across policy areas.

**Revised manuscript text (Page 11-12, Lines 368-408):**

“**Policy Implications**

Implications for policy and practice follow directly from the findings. First, evidence-informed policymaking (EIPM) should be institutionalised through the establishment or strengthening of national and subnational evidence synthesis units that can translate research into policy briefs, options papers, economic analyses, and implementation guidance [25]. Second, governance arrangements for EIPM should be strengthened by clarifying roles, mandates, and coordination mechanisms across sectors and levels of government, particularly for multisectoral policy areas such as non-communicable diseases (NCDs), nutrition, and climate-health [21,24,28]. Third, investments are needed in technical capacity and digital infrastructure, including training policymakers in evidence appraisal and equity assessment, strengthening health information systems, and promoting concise and accessible evidence products for decision-makers [20,26].

Fourth, research agendas should be aligned more closely with national and local policy priorities through structured priority-setting processes involving policymakers, implementers, researchers, and communities. Publicly funded research should be encouraged to demonstrate policy relevance and practical applicability, while co-funding approaches may help reduce dependence on donor-driven priorities [19,23]. Fifth, implementation science approaches should be used to translate policy intentions into actionable plans with clear indicators, budgets, timelines, and accountability mechanisms [26,30]. Sixth, transparency measures, conflict-of-interest policies, and public evidence hearings should be strengthened to safeguard policymaking from undue influence [19,27]. Finally, global frameworks such as WHO “Best Buys” and WHO-INTEGRATE should be adapted to Nepal's context and accompanied by clear operational guidance for implementation [21,35].

Given Nepal's ongoing federal transition, strengthening EIPM requires clearly defined and complementary roles across federal, provincial, and local governments. Federal institutions should provide strategic leadership through national evidence standards, research priority-setting, and integrated health information systems. Provincial governments should strengthen technical capacity, support cross-sectoral coordination, and adapt national policies to regional contexts, while local governments should be empowered to generate and use local data, engage communities in decision-making, and implement evidence-informed interventions tailored to local needs and socio-cultural realities. Strengthening accountability, communication, and coordination across all three levels of government is essential to address fragmentation, improve policy coherence, and ensure consistent use of evidence throughout the policy cycle.

Equity, contextualisation, and community engagement should remain central to these efforts. Evidence-aligned but context-insensitive policies may experience limited uptake and exacerbate inequities. Embedding equity assessments and meaningful stakeholder participation throughout agenda setting, policy formulation, implementation, and evaluation can strengthen legitimacy, ownership, and accountability. Similarly, investments in digital health architecture, including real-time reporting systems, decision-support tools, and community-based digital platforms, should be accompanied by workforce development and governance safeguards to ensure reliable and responsible use of evidence [19,32].”

7) *The abstract would read more smoothly if it were written as a single unstructured abstract rather than being divided into Background, Methods, Results, and Conclusions.*

Thank you for this suggestion. We appreciate that an unstructured abstract may improve narrative flow. However, we have retained the structured abstract format because it is widely used in PLOS ONE publications and provides readers with a clear presentation of the study background, methods, results, and conclusions. Nevertheless, we have revised the abstract text to improve readability and overall flow.

**Revised manuscript text (Page 2, Lines 25-54):**

**“Abstract**

**Background:** Evidence-informed health policymaking (EIPM) is essential for strengthening health systems and achieving equitable health outcomes. Despite policy commitments in Nepal, the translation of research evidence into policy and practice remains limited. This scoping review synthesizes the barriers and facilitators influencing EIPM across health policy domains in Nepal.

**Methods:** We conducted a scoping review following the Arksey and O'Malley framework and PRISMA-ScR guidelines. Peer-reviewed studies published between 2015 and 2025 that examined barriers and facilitators of EIPM in Nepal were identified through searches of PubMed, Scopus, Embase, and Global Health. Two reviewers independently screened studies, extracted data, and conducted descriptive and thematic analyses.

**Results:** Of 2,139 records identified, 26 studies met the inclusion criteria. The studies covered maternal and neonatal health, non-communicable diseases (NCDs), nutrition, emergency care, health systems governance, climate-health, public health regulation, and COVID-19 ethics. Barriers were grouped into five themes: systemic and governance challenges; capacity and resource constraints; evidence-related issues; contextual and cultural factors; and operational gaps. Common barriers included political interference, fragmented coordination, workforce shortages, inadequate funding, donor-driven agendas, poor dissemination of research, and limited implementation guidance. Facilitators clustered into four themes: institutional and structural enablers, political and leadership commitment, capacity building and stakeholder engagement, and global and donor support. Sector-specific differences were observed, with socio-cultural barriers more prominent in maternal and neonatal health, governance and coordination challenges particularly evident in NCD policies, and technical capacity and data limitations more pronounced in climate-health policy.

**Conclusions:** Nepal's EIPM landscape reflects strong commitment but uneven institutionalization. Strengthening governance, institutional capacity, evidence systems, and coordination across federal, provincial, and local institutions is essential to improve the translation of research into policy and practice. Institutionalizing EIPM requires not only producing evidence but also creating systems that routinely generate, translate, and use evidence for decision-making.”

***Reviewer #2:***

1) *First, the databases searched don't match across the paper. The abstract and the PRISMA diagram list PubMed, Scopus, Embase, and Global Health (with counts for each: 854, 127, 674, and 484), whereas the Methods section names PubMed, Scopus, Web of Science Core Collection, and NepJOL. These aren't equivalent resources, so the reader can't tell which search was actually run. Since the counts in Figure 1 line up with the Embase/Global Health version, my guess is that the Methods text is the one that wasn't updated — but the authors should confirm this and make the two sections agree. While they're at it, it would help to reconcile the search date (the text says October 2025, but Figure 1 is dated 16 December 2025) and to make sure the search-strategy appendix covers every database that was genuinely searched.*

Thank you for highlighting this issue. As noted in our response to the previous comment, we identified and corrected inconsistencies in the reporting of the search strategy across the manuscript. The Methods section has been revised to ensure consistency with the Abstract, PRISMA flow diagram, and Supplementary File 1 regarding the databases searched. We have also clarified the search dates and verified that the supplementary search strategies correspond to all databases included in the review.

**Revised manuscript text (Page 4, Lines 119-124):**

**“Information Sources and Search Strategy**

An electronic search was conducted in October 2025 through PubMed, Scopus, Embase, and Global Health using the keywords: evidence-informed, knowledge translation, policy making, barriers, challenges, facilitators, opportunities and Nepal. These keywords were searched in Medline using the Boolean operators “AND” and “OR” (a detailed search strategy of each database is provided in additional file 1).”

2) *Second, a few of the numbers in the flow don't add up. The Results refer to 2,139 "unique" records, but the diagram also reports 489 duplicates removed by Rayyan — records can't really be both unique and duplicated, so the wording needs a small fix. Of the 724 references removed before screening, only the 489 duplicates are explained, which leaves 235 without a stated reason. And the study-design breakdown (qualitative 18, mixed-methods 5, policy reviews 4, surveys 3) comes to 30 rather than 26. If some studies were counted under more than one design, just saying so would resolve it; otherwise these are worth correcting, since the record flow is really the backbone of a PRISMA-ScR review.,*

Thank you for these helpful observations. We have carefully reviewed the study selection process, PRISMA flow diagram, and reporting of study characteristics and have revised the manuscript accordingly. First, we corrected the wording in the Results section to indicate that 2,139 records were identified through database searching, rather than describing them as "unique" records, as duplicate records were subsequently removed. Second, we identified an error in the PRISMA flow diagram. A total of 724 duplicate records were removed prior to screening, and the figure has been revised accordingly. Third, we clarified that the study design categories were not mutually exclusive, as some included studies employed multiple methodological approaches and were therefore classified under more than one study design category. We have revised the Results section to explicitly state this and avoid potential confusion.

**Revised manuscript text (Page 4, Lines 159-175):**

“**Results**

A total of 2,139 records were identified through database searches. After the removal of 724 duplicate records, 1,415 records were screened by title and abstract. Following screening, 48 articles were retrieved and assessed for full-text eligibility. Of these, 22 articles were excluded for the following reasons: review articles without evidence used (n=6), protocol papers (n=2), synopsis article (n=1), absence of barriers or facilitators relevant to the review objective (n=4), no discussion of evidence-informed policies (n=4), and studies not conducted in Nepal (n=5). A total of 26 studies met the inclusion criteria and were included in the review. The complete study selection process is summarised in Figure 1. The highest number of studies was published in 2019, with a total of 5 studies, followed by 2020 with 4 studies. Years 2016, 2021, 2022, 2023, and 2025 each recorded 3 studies, indicating moderate activity (Figure 2).

**Study Characteristics**

The included studies spanned 2015–2025, reflecting growing interest in evidence-informed health policymaking (EIPM) in Nepal. Study designs were predominantly qualitative (n = 18), complemented by mixed-methods studies (n = 5), policy document reviews (n = 4), and quantitative surveys (n = 3). As some studies were classified into more than one study design category, the totals exceed the 26 studies included in the review.”

3) *Third, I'd encourage the authors to be a bit more explicit about what is genuinely Nepal-specific here. Quite a few of the included studies are multi-country LMIC papers in which Nepal is one case among several (Hipgrave et al., Buse et al., Witter et al., Binagwaho et al., and others). That's perfectly legitimate, but because the paper's headline contribution is a synthesis for Nepal, it would strengthen the argument to flag which barriers and facilitators rest on Nepal-specific observation versus those carried over from cross-national work — perhaps as a column in Supplementary Table 1. Where a theme leans mostly on multi-country evidence, the corresponding claims could be stated a little more cautiously.*

Thank you for this comment. We agree that distinguishing between Nepal-specific findings and those derived from multi-country studies strengthens the interpretation of the review. We have updated Supplementary Table 1 to indicate whether studies were conducted exclusively in Nepal or as part of a multi-country analysis. In the Results section, we now explicitly report that most included studies were conducted exclusively in Nepal, while a smaller number (n = 6) were multi-country studies that included Nepal as one of the country cases. In addition, we have acknowledged this issue in the Strengths and Limitations section by noting that some included studies were multi-country analyses and that their findings should be interpreted alongside Nepal-specific evidence.

**Revised manuscript text in Results (Page 7, Lines 207-209):**

Most included studies were conducted exclusively in Nepal, while a smaller number (n=6) were multi-country studies that included Nepal as one of the country cases. Findings from both study types contributed to the thematic synthesis.

**Revised manuscript text in Strengths and Limitations (Page 12-13, Lines 417-420):**

Some included studies were multi-country analyses that included Nepal as one of the country cases. Therefore, certain barriers and facilitators may reflect broader LMIC experiences rather than challenges unique to Nepal, although findings were interpreted alongside Nepal-specific evidence.

4) *Finally, two smaller points that I've grouped together because they're related to scope. The Discussion and Policy Implications occasionally read more prescriptively than the underlying evidence supports — recommendations built around RE-AIM, CFIR, equity impact assessments, and the like sometimes feel like the authors' own (reasonable) views rather than findings traceable to the 26 included studies. Given that a scoping review is meant to map the literature rather than appraise or prescribe, I'd gently suggest dialing back the prescriptive tone and tying each recommendation to its source where possible. On a similar note, the protocol is said to be registered with PROSPERO, but PROSPERO generally doesn't accept scoping reviews, and the link points to a PDF rather than a registration record. It would be worth clarifying the registration status, and if PROSPERO doesn't apply, an alternative such as OSF might be more appropriate.*

Thank you for raising these points. We agree that, as a scoping review, the primary purpose of this study is to map and synthesise the existing literature rather than prescribe specific interventions. As noted in our response to the previous comment on policy implications, we have reviewed the Policy Implications and Recommendations for Future Research sections and revised the language to ensure that recommendations are more closely aligned with the evidence identified in the included studies. We have also clarified the status of the review protocol. Although a protocol was developed prior to conducting the review, it was not formally registered with PROSPERO. We have therefore revised the manuscript by removing references implying PROSPERO registration and clarifying that the protocol was developed a priori but was not formally registered.

**Revised manuscript text for Protocol and registration (Page 5, Lines 156-158):**

**Protocol and registration:** A protocol was developed a priori to guide the conduct of this scoping review; however, it was not formally registered. <https://www.crd.york.ac.uk/PROSPEROFILES/9c4342e8fafe7937132689bc020ba9df.pdf>

**Revised manuscript text (Page 11-12, Lines 368-408)**

**Policy Implications**

Implications for policy and practice follow directly from the findings. First, evidence-informed policymaking (EIPM) should be institutionalised through the establishment or strengthening of national and subnational evidence synthesis units that can translate research into policy briefs, options papers, economic analyses, and implementation guidance [25]. Second, governance arrangements for EIPM should be strengthened by clarifying roles, mandates, and coordination mechanisms across sectors and levels of government, particularly for multisectoral policy areas such as non-communicable diseases (NCDs), nutrition, and climate-health [24,21,28]. Third, investments are needed in technical capacity and digital infrastructure, including training policymakers in evidence appraisal and equity assessment, strengthening health information systems, and promoting concise and accessible evidence products for decision-makers [26,20].

Fourth, research agendas should be aligned more closely with national and local policy priorities through structured priority-setting processes involving policymakers, implementers, researchers, and communities. Publicly funded research should be encouraged to demonstrate policy relevance and practical applicability, while co-funding approaches may help reduce dependence on donor-driven priorities [19,23]. Fifth, implementation science approaches should be used to translate policy intentions into actionable plans with clear indicators, budgets, timelines, and accountability mechanisms [26,30]. Sixth, transparency measures, conflict-of-interest policies, and public evidence hearings should be strengthened to safeguard policymaking from undue influence [19,27]. Finally, global frameworks such as WHO “Best Buys” and WHO-INTEGRATE should be adapted to Nepal's context and accompanied by clear operational guidance for implementation [21,35].

Given Nepal's ongoing federal transition, strengthening EIPM requires clearly defined and complementary roles across federal, provincial, and local governments. Federal institutions should provide strategic leadership through national evidence standards, research priority-setting, and integrated health information systems. Provincial governments should strengthen technical capacity, support cross-sectoral coordination, and adapt national policies to regional contexts, while local governments should be empowered to generate and use local data, engage communities in decision-making, and implement evidence-informed interventions tailored to local needs and socio-cultural realities. Strengthening accountability, communication, and coordination across all three levels of government is essential to address fragmentation, improve policy coherence, and ensure consistent use of evidence throughout the policy cycle.

Equity, contextualisation, and community engagement should remain central to these efforts. Evidence-aligned but context-insensitive policies may experience limited uptake and exacerbate inequities. Embedding equity assessments and meaningful stakeholder participation throughout agenda setting, policy formulation, implementation, and evaluation can strengthen legitimacy, ownership, and accountability. Similarly, investments in digital health architecture, including real-time reporting systems, decision-support tools, and community-based digital platforms, should be accompanied by workforce development and governance safeguards to ensure reliable and responsible use of evidence [19,32].”

**Revised manuscript text (Page 13, Lines 421-431):**

**Recommendations for Future Research**

Future research should focus on developing and evaluating approaches to assess evidence-informed policymaking across federal, provincial, and local levels in Nepal. Additional studies are needed to evaluate the effectiveness of evidence synthesis mechanisms, policy dialogues, and other knowledge translation strategies in supporting policy decisions. Comparative research exploring different approaches to evidence dissemination and stakeholder engagement may help identify contextually appropriate strategies for promoting evidence use in policymaking. Further research is also needed to examine governance, financing, and implementation challenges in priority policy areas, including non-communicable diseases, maternal and neonatal health, nutrition, and climate-health, within Nepal's federal health system.

5) *A few minor things I noticed:

   (i) References 6 and 31 (both Tiwari et al., BMC Health Serv Res, 2021) look like the same paper entered twice and cited under both numbers; these should be merged.*

*(ii) Some citations seem mismatched to the text. The passage on food-marketing restrictions and private-sector interference cites 27 (Gaihre et al., on nutrition/food security), but the content really points to Fisher et al. (37). The placement of Stratil (36) and Namirembe (26) under "Contextual and Cultural Factors" in the tables also reads a little oddly. A pass over the citation-to-claim and table-to-text mapping would be worthwhile.

(iii) Reference numbering doesn't follow order of first appearance (e.g., "24, 16, 19, 20, 26"); this will need renumbering for PLOS style.

(iv) The text describing Figure 2 skips 2015 and 2024 (each n=1), so the figure and the narrative aren't quite in step.

(v) I didn't see any inter-rater agreement statistic for screening or coding. It isn't strictly required for a scoping review, but reporting one would add confidence in the synthesis.

(vi) Reference 15 (PRISMA-ScR, "Tricco* [*et.al*](http://et.al/)*.") has incomplete authorship and some formatting issues; a general reference check would catch this and a few similar cases.*

Thank you for these careful and constructive observations. We have addressed each of these points during revision.

(i) We identified that References 6 and 31 referred to the same publication (Tiwari et al., 2021) and have removed the duplicate entry and updated the corresponding citations throughout the manuscript.
(ii) We reviewed all in-text citations, tables, and references to ensure that cited studies accurately support the associated statements. We corrected citation mismatches, including the citation relating to food-marketing restrictions and private-sector interference, and reviewed the allocation of references within the thematic tables to improve consistency between findings and supporting evidence.
(iii) We checked and updated reference numbering to ensure compliance with journal formatting requirements and consistency with the order of first citation in the text.
(iv) We revised the description of Figure 2 to ensure consistency with the figure and to account for studies published in 2015 and 2024.

**Revised manuscript text (Page 6, Lines 167-170):**

The highest number of studies was published in 2019 (n = 5), followed by 2020 (n = 4). The years 2016, 2021, 2022, 2023, and 2025 each contributed three studies, while 2015 and 2024 each contributed one study (Figure 2).

(v) We acknowledge that an inter-rater agreement statistic was not calculated during screening or coding. While such statistics are not required for scoping reviews, we have clarified that screening, data extraction, and thematic coding were undertaken independently by two reviewers, with discrepancies resolved through discussion and consensus.
(vi) We have reviewed and corrected the reference list, including the PRISMA-ScR citation. Reference 15 has been updated to include the complete authorship and correct bibliographic details. We also conducted a comprehensive review of all references and in-text citations to ensure consistency, accuracy, and compliance with the journal's formatting requirements. The corrected reference could be presented as:

“Tricco AC, Lillie E, Zarin W, O'Brien KK, Colquhoun H, Levac D, et al. PRISMA extension for scoping reviews (PRISMA-ScR): checklist and explanation. Annals of Internal Medicine. 2018;169(7):467-473. doi:10.7326/M18-0850”

*The keywords metadata field appears blank even though keywords are given in the manuscript itself.*

Thank you for pointing this out. The keywords were included in the manuscript; however, it appears that the keywords metadata field was not populated correctly. We have now uploaded it as a PDF and ensured that the keywords are clearly included.
