# Supplementary material for: Barriers and facilitators of evidence-informed health policymaking in Nepal: A scoping review
Source: PLoS One. 2026 Oct 26;21(10):e0358730. doi: 10.1371/journal.pone.0358730 (PMC13704804; doi:10.1371/journal.pone.0358730)
Supplement: Supplementary file 5 [file pone.0358730.s006.docx]

Editorial Team

PLOS One

22^nd^ September 2026

**Re: Manuscript reference number-PONE-D-26-00588R1**

Dear Editorial Team,

I am writing to submit a revision of the above manuscript. We sincerely appreciate the helpful comments from the reviewer, and we have revised the manuscript according to his/her suggestions.

The modified sections in the manuscript are highlighted using tracked changes. Please find the attached response to the reviewer which details the changes we have made along with explanations for these changes.

We hope that the revised manuscript will now be acceptable for publication in *PLOS One* and look forward to your decision. Thank you very much for your consideration of this manuscript.

Yours sincerely,

Dr Kaung Suu Lwin, MBBS MHSc PhD

School of Human and Health Sciences

University of Huddersfield | Queensgate | Huddersfield | HD1 3DH

**RESPONSE TO REVIEWERS**

We would like to thank the editor and reviewer for providing helpful comments. Our responses are given beneath each comment. The added/revised text is double-quoted in our response for ease of reference, with line and page numbers provided where necessary.

***Reviewer #2:***

1) *Protocol registration statement (lines 157-159). The text now correctly states that the protocol "was not formally registered," but the PROSPERO URL is retained immediately below. This is internally contradictory and may mislead readers into assuming a registration record exists. Please either remove the link or reframe it (e.g., "The a priori protocol document is available at ...") and consider depositing it in a suitable repository such as OSF.*

Thank you for this important observation. We agree that the previous wording could create confusion by implying the existence of a formal PROSPERO registration. To address this, we have revised the manuscript to clarify that the review protocol was developed a priori but was not formally registered. We have removed the PROSPERO link and deposited the protocol in the Open Science Framework (OSF).

The revised text now reads:

"The review protocol was developed a priori but was not formally registered. To enhance transparency and accessibility, the protocol has been deposited in the Open Science Framework (OSF) and is available at: <https://osf.io/fp27w>." (Page 5, Lines 157-159)

2) *Table 1, Contextual and Cultural Factors. Stratil et al. [35] and Namirembe et al. [26] remain listed under this theme. The response letter states that the allocation of references in the thematic tables was reviewed, but no change appears to have been made here. Please confirm that these placements are intentional; on my reading, both studies fit more naturally under governance or evidence-related themes.*

Thank you for this helpful observation. We have reviewed the placement of Stratil et al. [35] and Namirembe et al. [26] and confirm that their allocation under the Contextual and Cultural Factors theme is intentional. Although both studies discuss governance and evidence-use issues, they also highlight the influence of contextual, social, and cultural environments on the translation and uptake of evidence in policymaking. Because themes were not treated as mutually exclusive during synthesis, we retained these studies within the contextual and cultural domain.

3) *Possible duplicate reference. Reference 8 (Dhimal et al., 2016, J Nepal Health Res Counc, "Utilization of health research recommendation in policy and planning in Nepal") and Reference 31 (Dhimal et al., 2016, NHRC report, "Translation of health research evidence into policy and planning in Nepal: an appraisal 2016") appear to describe the same body of work. Please verify whether these are distinct sources; if not, they should be merged.*

Thank you for identifying this potential duplication. We reviewed both references and confirmed that they are distinct sources, although they arise from the same body of work and share several authors.

Reference 8 is a peer-reviewed journal article published in the Journal of Nepal Health Research Council:

Dhimal M, Pandey AR, Aryal KK, Budhathoki CB, Vaidya DL, Karki KK, et al. Utilization of health research recommendation in policy and planning in Nepal. J Nepal Health Res Counc. 2016;14(34):165-172.

Reference 31 is a technical report published by the Nepal Health Research Council (NHRC):

Dhimal M, Pandey AR, Aryal KK, Subedi M, Karki KB. Translation of health research evidence into policy and planning in Nepal: an appraisal 2016. Kathmandu: Nepal Health Research Council; 2016.

While both publications address the translation and utilization of health research evidence in policy and planning in Nepal, they represent different publication formats and contain differing levels of detail. The NHRC report provides a more comprehensive institutional appraisal, whereas the journal article presents a peer-reviewed synthesis of key findings. We have therefore retained both references and have reviewed the relevant citations in the manuscript to ensure that each source is cited appropriately and for its distinct contribution.

4) *Funding statement. I note that the funding statement has changed from "no external funding" in the original submission to British Academy support in the revision. This is not a matter for peer review, but the editorial office may wish to confirm the change is intended.*

Thank you for noting this change. The revised funding statement is correct and the change was intentional. During preparation of the revised manuscript, we identified that funding support provided by the British Academy had been inadvertently omitted from the original submission. To ensure transparency and compliance with the journal's funding disclosure requirements, the funding statement has been corrected to accurately acknowledge this support.

5) *Minor typographical points. Line 241, "weak-evidence" should read "weak—evidence" (em dash). Line 123, "Medline," as noted above.*

Thank you for noting these typographical errors. We have corrected "weak-evidence" to "weak— evidence" (Line 241) and revised "Medline" to "MEDLINE" (Line 123).
