# Supplementary material for: Barriers and facilitators of evidence-informed health policymaking in Nepal: A scoping review
Source: PLoS One. 2026 Oct 26;21(10):e0358730. doi: 10.1371/journal.pone.0358730 (PMC13704804; doi:10.1371/journal.pone.0358730)
Supplement: Peer review history

# Peer review: Decision Letter 0

**DOI:** 10.1371/journal.pone.0358730.r001

**Authors:** Kshitij Karki

30 Jun 2026

PONE-D-26-00588Barriers and facilitators of evidence-informed health policymaking in Nepal: a scoping reviewPLOS One

Dear Dr. Kaung Suu Lwin,

Thank you for submitting your manuscript to PLOS ONE. After careful consideration, we feel that it has merit but does not fully meet PLOS ONE’s publication criteria as it currently stands. Therefore, we invite you to submit a revised version of the manuscript that addresses the points raised during the review process.

Please address all the comments from the reviewers.  Please submit your revised manuscript by  Aug 14 2026 11:59PM. If you will need more time than this to complete your revisions, please reply to this message or contact the journal office at plosone@plos.org. When you're ready to submit your revision, log on to https://www.editorialmanager.com/pone/ and select the 'Submissions Needing Revision' folder to locate your manuscript file.

Please include the following items when submitting your revised manuscript:

- A letter that responds to each point raised by the academic editor and reviewer(s). You should upload this letter as a separate file labeled 'Response to Reviewers'.
- A marked-up copy of your manuscript that highlights changes made to the original version. You should upload this as a separate file labeled 'Revised Manuscript with Track Changes'.
- An unmarked version of your revised paper without tracked changes. You should upload this as a separate file labeled 'Manuscript'.

If you would like to make changes to your financial disclosure, please include your updated statement in your cover letter. Guidelines for resubmitting your figure files are available below the reviewer comments at the end of this letter.

If applicable, we recommend that you deposit your laboratory protocols in protocols.io to enhance the reproducibility of your results. Protocols.io assigns your protocol its own identifier (DOI) so that it can be cited independently in the future. For instructions see: https://journals.plos.org/plosone/s/submission-guidelines#loc-laboratory-protocols. Additionally, PLOS ONE offers an option for publishing peer-reviewed Lab Protocol articles, which describe protocols hosted on protocols.io. Read more information on sharing protocols at https://plos.org/protocols?utm_medium=editorial-email&utm_source=authorletters&utm_campaign=protocols.

As the corresponding author, your ORCID iD is verified in the submission system and will appear in the published article. PLOS supports the use of ORCID, and we encourage all coauthors to register for an ORCID iD and use it as well. Please encourage your coauthors to verify their ORCID iD within the submission system before final acceptance, as unverified ORCID iDs will not appear in the published article. *Only*  the individual author can complete the verification step; PLOS staff *cannot*  verify ORCID iDs on behalf of authors.

We look forward to receiving your revised manuscript.

Kind regards,

Kshitij Karki, MPH, MA

Academic Editor

PLOS One

Journal Requirements:

When submitting your revision, we need you to address these additional requirements.

1. Please ensure that your manuscript meets PLOS ONE's style requirements, including those for file naming. The PLOS ONE style templates can be found at

https://journals.plos.org/plosone/s/file?id=wjVg/PLOSOne_formatting_sample_main_body.pdf and

https://journals.plos.org/plosone/s/file?id=ba62/PLOSOne_formatting_sample_title_authors_affiliations.pdf

2. PLOS requires an ORCID iD for the corresponding author in Editorial Manager on papers submitted after December 6th, 2016. Please ensure that you have an ORCID iD and that it is validated in Editorial Manager. To do this, go to ‘Update my Information’ (in the upper left-hand corner of the main menu), and click on the Fetch/Validate link next to the ORCID field. This will take you to the ORCID site and allow you to create a new iD or authenticate a pre-existing iD in Editorial Manager.

3. Please include captions for your Supporting Information files at the end of your manuscript, and update any in-text citations to match accordingly. Please see our Supporting Information guidelines for more information: http://journals.plos.org/plosone/s/supporting-information.

4. If the reviewer comments include a recommendation to cite specific previously published works, please review and evaluate these publications to determine whether they are relevant and should be cited. There is no requirement to cite these works unless the editor has indicated otherwise.

Additional Editor Comments:

Please go through the comments from the reviewers and address them.

[Note: HTML markup is below. Please do not edit.]

Reviewers' comments:

Reviewer's Responses to Questions

**Comments to the Author**

1. Is the manuscript technically sound, and do the data support the conclusions?

The manuscript must describe a technically sound piece of scientific research with data that supports the conclusions. Experiments must have been conducted rigorously, with appropriate controls, replication, and sample sizes. The conclusions must be drawn appropriately based on the data presented.

Reviewer #1: Partly

Reviewer #2: Partly

**********

2. Has the statistical analysis been performed appropriately and rigorously?

Reviewer #1: I Don't Know

Reviewer #2: No

**********

3. Have the authors made all data underlying the findings in their manuscript fully available?

The PLOS Data policy requires authors to make all data underlying the findings described in their manuscript fully available without restriction, with rare exception (please refer to the Data Availability Statement in the manuscript PDF file). The data should be provided as part of the manuscript or its supporting information, or deposited to a public repository. For example, in addition to summary statistics, the data points behind means, medians and variance measures should be available. If there are restrictions on publicly sharing data—e.g. participant privacy or use of data from a third party—those must be specified.

Reviewer #1: No

Reviewer #2: Yes

**********

4. Is the manuscript presented in an intelligible fashion and written in standard English?

PLOS ONE does not copyedit accepted manuscripts, so the language in submitted articles must be clear, correct, and unambiguous. Any typographical or grammatical errors should be corrected at revision, so please note any specific errors here.

Reviewer #1: Yes

Reviewer #2: Yes

**********

5. Review Comments to the Author

Please use the space provided to explain your answers to the questions above. You may also include additional comments for the author, including concerns about dual publication, research ethics, or publication ethics. (Please upload your review as an attachment if it exceeds 20,000 characters)

Reviewer #1: - The search strategy is reported inconsistently. The abstract states that searches were conducted in PubMed, Scopus, Embase, and Global Health, whereas the Methods section lists PubMed, Scopus, Web of Science Core Collection, and NepJOL. The authors should clarify which databases were actually searched.

- The PRISMA-ScR reporting should be strengthened. The manuscript reports that 2,139 records were identified, 48 full-text articles were screened, and 26 studies were included, but the screening and exclusion process is not sufficiently detailed. The authors should provide clearer information on duplicate removal, full-text exclusion reasons, and the study selection process.

- The manuscript states that 26 studies were included, but the numbers reported by study design add up to 30: qualitative studies (n=18), mixed-methods studies (n=5), policy document reviews (n=4), and quantitative surveys (n=3). The authors should clarify whether these categories are mutually exclusive.

- The thematic synthesis needs to be described more transparently. The manuscript states that text segments related to barriers and facilitators were coded and grouped into broader categories and overarching themes. However, it does not provide enough detail on what coding framework was used, how inductive coding was conducted, how disagreements between reviewers were resolved, and how the final themes were derived.

- The authors should further develop the sector-specific interpretation of the findings. For example, it would be useful to explain whether certain barriers, such as donor-driven agendas, weak governance, or local socio-cultural norms, are more pronounced in specific policy areas such as non-communicable diseases, maternal and neonatal health, nutrition, or climate-health policy.

- The policy implications should be made more specific. Many of the identified barriers and facilitators are common across low- and middle-income countries. The manuscript would be stronger if it explained more clearly how Nepal’s federalized health governance system shapes these barriers and what roles should be played by national, provincial, and local institutions.

- The abstract would read more smoothly if it were written as a single unstructured abstract rather than being divided into Background, Methods, Results, and Conclusions.

Reviewer #2: I enjoyed reading this manuscript. Evidence-informed policymaking in Nepal is an important and under-synthesized topic, and as far as I can tell the authors are right that no prior scoping review has pulled this literature together. The thematic structure is sensible, the writing is generally clear, and the policy framing will be useful to readers working in this space. My reservations are not about the value of the work but about a handful of reporting inconsistencies that, as the manuscript stands, would make it hard for a reader to fully trust or reproduce the search and synthesis. I think these are fixable, and I'd be glad to see a revised version.

My main concerns are the following.

First, the databases searched don't match across the paper. The abstract and the PRISMA diagram list PubMed, Scopus, Embase, and Global Health (with counts for each: 854, 127, 674, and 484), whereas the Methods section names PubMed, Scopus, Web of Science Core Collection, and NepJOL. These aren't equivalent resources, so the reader can't tell which search was actually run. Since the counts in Figure 1 line up with the Embase/Global Health version, my guess is that the Methods text is the one that wasn't updated — but the authors should confirm this and make the two sections agree. While they're at it, it would help to reconcile the search date (the text says October 2025, but Figure 1 is dated 16 December 2025) and to make sure the search-strategy appendix covers every database that was genuinely searched.

Second, a few of the numbers in the flow don't add up. The Results refer to 2,139 "unique" records, but the diagram also reports 489 duplicates removed by Rayyan — records can't really be both unique and duplicated, so the wording needs a small fix. Of the 724 references removed before screening, only the 489 duplicates are explained, which leaves 235 without a stated reason. And the study-design breakdown (qualitative 18, mixed-methods 5, policy reviews 4, surveys 3) comes to 30 rather than 26. If some studies were counted under more than one design, just saying so would resolve it; otherwise these are worth correcting, since the record flow is really the backbone of a PRISMA-ScR review.

Third, I'd encourage the authors to be a bit more explicit about what is genuinely Nepal-specific here. Quite a few of the included studies are multi-country LMIC papers in which Nepal is one case among several (Hipgrave et al., Buse et al., Witter et al., Binagwaho et al., and others). That's perfectly legitimate, but because the paper's headline contribution is a synthesis for Nepal, it would strengthen the argument to flag which barriers and facilitators rest on Nepal-specific observation versus those carried over from cross-national work — perhaps as a column in Supplementary Table 1. Where a theme leans mostly on multi-country evidence, the corresponding claims could be stated a little more cautiously.

Finally, two smaller points that I've grouped together because they're related to scope. The Discussion and Policy Implications occasionally read more prescriptively than the underlying evidence supports — recommendations built around RE-AIM, CFIR, equity impact assessments, and the like sometimes feel like the authors' own (reasonable) views rather than findings traceable to the 26 included studies. Given that a scoping review is meant to map the literature rather than appraise or prescribe, I'd gently suggest dialing back the prescriptive tone and tying each recommendation to its source where possible. On a similar note, the protocol is said to be registered with PROSPERO, but PROSPERO generally doesn't accept scoping reviews, and the link points to a PDF rather than a registration record. It would be worth clarifying the registration status, and if PROSPERO doesn't apply, an alternative such as OSF might be more appropriate.

A few minor things I noticed:

References 6 and 31 (both Tiwari et al., BMC Health Serv Res, 2021) look like the same paper entered twice and cited under both numbers; these should be merged.

Some citations seem mismatched to the text. The passage on food-marketing restrictions and private-sector interference cites 27 (Gaihre et al., on nutrition/food security), but the content really points to Fisher et al. (37). The placement of Stratil (36) and Namirembe (26) under "Contextual and Cultural Factors" in the tables also reads a little oddly. A pass over the citation-to-claim and table-to-text mapping would be worthwhile.

Reference numbering doesn't follow order of first appearance (e.g., "24, 16, 19, 20, 26"); this will need renumbering for PLOS style.

The text describing Figure 2 skips 2015 and 2024 (each n=1), so the figure and the narrative aren't quite in step.

I didn't see any inter-rater agreement statistic for screening or coding. It isn't strictly required for a scoping review, but reporting one would add confidence in the synthesis.

Reference 15 (PRISMA-ScR, "Tricco et.al.") has incomplete authorship and some formatting issues; a general reference check would catch this and a few similar cases.

The keywords metadata field appears blank even though keywords are given in the manuscript itself.

**********

6. PLOS authors have the option to publish the peer review history of their article (what does this mean?). If published, this will include your full peer review and any attached files.

If you choose “no”, your identity will remain anonymous but your review may still be made public.

**Do you want your identity to be public for this peer review?**  For information about this choice, including consent withdrawal, please see our Privacy Policy.

Reviewer #1: No

Reviewer #2: **Yes:**  Jisung Yoon

**********

[NOTE: If reviewer comments were submitted as an attachment file, they will be attached to this email and accessible via the submission site. Please log into your account, locate the manuscript record, and check for the action link "View Attachments". If this link does not appear, there are no attachment files.]

To ensure your figures meet our technical requirements, please review our figure guidelines: https://journals.plos.org/plosone/s/figures

You may also use PLOS’s free figure tool, NAAS, to help you prepare publication quality figures: https://journals.plos.org/plosone/s/figures#loc-tools-for-figure-preparation.

NAAS will assess whether your figures meet our technical requirements by comparing each figure against our figure specifications.

# Author comment: Author response to Decision Letter 1

**DOI:** 10.1371/journal.pone.0358730.r002

24 Aug 2026

Dear Editorial Team,

I am writing to submit a revision of the above manuscript. We sincerely appreciate the helpful comments from the reviewer, and we have revised the manuscript according to his/her suggestions.

The modified sections in the manuscript are highlighted using tracked changes. Please find the attached response to the reviewer which details the changes we have made along with explanations for these changes.

We hope that the revised manuscript will now be acceptable for publication in PLOS One and look forward to your decision. Thank you very much for your consideration of this manuscript.

Yours sincerely,

Dr Kaung Suu Lwin, MBBS MHSc PhD

School of Human and Health Sciences

University of Huddersfield | Queensgate | Huddersfield | HD1 3DH

Attachment Submitted filename: Response letter_EIPM 13 Aug 2026.docx

# Peer review: Decision Letter 1

**DOI:** 10.1371/journal.pone.0358730.r003

**Authors:** Kshitij Karki

21 Sep 2026

PONE-D-26-00588R1Barriers and facilitators of evidence-informed health policymaking in Nepal: a scoping reviewPLOS One

Dear Dr. Lwin,

Thank you for submitting your manuscript to PLOS One. After careful consideration, we feel that it has merit but does not fully meet PLOS One’s publication criteria as it currently stands. Therefore, we invite you to submit a revised version of the manuscript that addresses the points raised during the review process.

Please address the comments from the reviewer.

Please submit your revised manuscript by Nov 05 2026 11:59PM. If you will need more time than this to complete your revisions, please reply to this message or contact the journal office at plosone@plos.org. When you're ready to submit your revision, log on to https://www.editorialmanager.com/pone/ and select the 'Submissions Needing Revision' folder to locate your manuscript file.

Please include the following items when submitting your revised manuscript:

- A letter that responds to each point raised by the academic editor and reviewer(s). You should upload this letter as a separate file labeled 'Response to Reviewers'.
- A marked-up copy of your manuscript that highlights changes made to the original version. You should upload this as a separate file labeled 'Revised Manuscript with Track Changes'.
- An unmarked version of your revised paper without tracked changes. You should upload this as a separate file labeled 'Manuscript'.

If you would like to make changes to your financial disclosure, please include your updated statement in your cover letter. Guidelines for resubmitting your figure files are available below the reviewer comments at the end of this letter.

If applicable, we recommend that you deposit your laboratory protocols in protocols.io to enhance the reproducibility of your results. Protocols.io assigns your protocol its own identifier (DOI) so that it can be cited independently in the future. For instructions see: https://journals.plos.org/plosone/s/submission-guidelines#loc-laboratory-protocols. Additionally, PLOS One offers an option for publishing peer-reviewed Lab Protocol articles, which describe protocols hosted on protocols.io. Read more information on sharing protocols at https://plos.org/protocols?utm_medium=editorial-email&utm_source=authorletters&utm_campaign=protocols.

As the corresponding author, your ORCID iD is verified in the submission system and will appear in the published article. PLOS supports the use of ORCID, and we encourage all coauthors to register for an ORCID iD and use it as well. Please encourage your coauthors to verify their ORCID iD within the submission system before final acceptance, as unverified ORCID iDs will not appear in the published article. *Only*  the individual author can complete the verification step; PLOS staff *cannot*  verify ORCID iDs on behalf of authors.

We look forward to receiving your revised manuscript.

Kind regards,

Kshitij Karki, MPH, MA

Academic Editor

PLOS One

Journal Requirements:

1. If the reviewer comments include a recommendation to cite specific previously published works, please review and evaluate these publications to determine whether they are relevant and should be cited. There is no requirement to cite these works unless the editor has indicated otherwise.

2. Please review your reference list to ensure that it is complete and correct. If you have cited papers that have been retracted, please include the rationale for doing so in the manuscript text, or remove these references and replace them with relevant current references. Any changes to the reference list should be mentioned in the rebuttal letter that accompanies your revised manuscript. If you need to cite a retracted article, indicate the article’s retracted status in the References list and also include a citation and full reference for the retraction notice.

**Additional Editor Comments:**

Please go through the comments from reviewers and address.

Thank you

[Note: HTML markup is below. Please do not edit.]

Reviewers' comments:

Reviewer's Responses to Questions

**Comments to the Author**

1. If the authors have adequately addressed your comments raised in a previous round of review and you feel that this manuscript is now acceptable for publication, you may indicate that here to bypass the “Comments to the Author” section, enter your conflict of interest statement in the “Confidential to Editor” section, and submit your "Accept" recommendation.

Reviewer #2: (No Response)

**********

2. Is the manuscript technically sound, and do the data support the conclusions?

The manuscript must describe a technically sound piece of scientific research with data that supports the conclusions. Experiments must have been conducted rigorously, with appropriate controls, replication, and sample sizes. The conclusions must be drawn appropriately based on the data presented.

Reviewer #2: Yes

**********

3. Has the statistical analysis been performed appropriately and rigorously?

Reviewer #2: Yes

**********

4. Have the authors made all data underlying the findings in their manuscript fully available?

The PLOS Data policy requires authors to make all data underlying the findings described in their manuscript fully available without restriction, with rare exception (please refer to the Data Availability Statement in the manuscript PDF file). The data should be provided as part of the manuscript or its supporting information, or deposited to a public repository. For example, in addition to summary statistics, the data points behind means, medians and variance measures should be available. If there are restrictions on publicly sharing data—e.g. participant privacy or use of data from a third party—those must be specified.

Reviewer #2: Yes

**********

5. Is the manuscript presented in an intelligible fashion and written in standard English?

PLOS ONE does not copyedit accepted manuscripts, so the language in submitted articles must be clear, correct, and unambiguous. Any typographical or grammatical errors should be corrected at revision, so please note any specific errors here.

Reviewer #2: Yes

**********

6. Review Comments to the Author

Please use the space provided to explain your answers to the questions above. You may also include additional comments for the author, including concerns about dual publication, research ethics, or publication ethics. (Please upload your review as an attachment if it exceeds 20,000 characters)

Reviewer #2: I thank the authors for their careful and well-organised response. The majority of my concerns have been addressed, and the manuscript is substantially improved. My point-by-point assessment follows, together with a small number of remaining issues, most of which are minor.

Additional issues noted in the revised manuscript

Protocol registration statement (lines 157-159). The text now correctly states that the protocol "was not formally registered," but the PROSPERO URL is retained immediately below. This is internally contradictory and may mislead readers into assuming a registration record exists. Please either remove the link or reframe it (e.g., "The a priori protocol document is available at ...") and consider depositing it in a suitable repository such as OSF.

Table 1, Contextual and Cultural Factors. Stratil et al. [35] and Namirembe et al. [26] remain listed under this theme. The response letter states that the allocation of references in the thematic tables was reviewed, but no change appears to have been made here. Please confirm that these placements are intentional; on my reading, both studies fit more naturally under governance or evidence-related themes.

Possible duplicate reference. Reference 8 (Dhimal et al., 2016, J Nepal Health Res Counc, "Utilization of health research recommendation in policy and planning in Nepal") and Reference 31 (Dhimal et al., 2016, NHRC report, "Translation of health research evidence into policy and planning in Nepal: an appraisal 2016") appear to describe the same body of work. Please verify whether these are distinct sources; if not, they should be merged.

Funding statement. I note that the funding statement has changed from "no external funding" in the original submission to British Academy support in the revision. This is not a matter for peer review, but the editorial office may wish to confirm the change is intended.

Minor typographical points. Line 241, "weak-evidence" should read "weak—evidence" (em dash). Line 123, "Medline," as noted above.

**********

7. PLOS authors have the option to publish the peer review history of their article (what does this mean?). If published, this will include your full peer review and any attached files.

If you choose “no”, your identity will remain anonymous but your review may still be made public.

**Do you want your identity to be public for this peer review?**  For information about this choice, including consent withdrawal, please see our Privacy Policy.

Reviewer #2: **Yes:**  Jisung Yoon

**********

[NOTE: If reviewer comments were submitted as an attachment file, they will be attached to this email and accessible via the submission site. Please log into your account, locate the manuscript record, and check for the action link "View Attachments". If this link does not appear, there are no attachment files.]

To ensure your figures meet our technical requirements, please review our figure guidelines: https://journals.plos.org/plosone/s/figures

You may also use PLOS’s free figure tool, NAAS, to help you prepare publication quality figures: https://journals.plos.org/plosone/s/figures#loc-tools-for-figure-preparation.

NAAS will assess whether your figures meet our technical requirements by comparing each figure against our figure specifications.

# Author comment: Author response to Decision Letter 2

**DOI:** 10.1371/journal.pone.0358730.r004

23 Sep 2026

Editorial Team

PLOS One

22nd September 2026

Re: Manuscript reference number-PONE-D-26-00588R1

Dear Editorial Team,

I am writing to submit a revision of the above manuscript. We sincerely appreciate the helpful comments from the reviewer, and we have revised the manuscript according to his/her suggestions.

The modified sections in the manuscript are highlighted using tracked changes. Please find the attached response to the reviewer which details the changes we have made along with explanations for these changes.

We hope that the revised manuscript will now be acceptable for publication in PLOS One and look forward to your decision. Thank you very much for your consideration of this manuscript.

Yours sincerely,

Dr Kaung Suu Lwin, MBBS MHSc PhD

School of Human and Health Sciences

University of Huddersfield | Queensgate | Huddersfield | HD1 3DH

Attachment Submitted filename: Response letter_EIPM 22 September 2026.docx

# Peer review: Decision Letter 2

**DOI:** 10.1371/journal.pone.0358730.r005

**Authors:** Kshitij Karki

7 Oct 2026

Barriers and facilitators of evidence-informed health policymaking in Nepal: a scoping review

PONE-D-26-00588R2

Dear Dr. Kaung Suu Lwin,

We’re pleased to inform you that your manuscript has been judged scientifically suitable for publication and will be formally accepted for publication once it meets all outstanding technical requirements.

Within one week, you’ll receive an e-mail detailing the required amendments. When these have been addressed, you’ll receive a formal acceptance letter and your manuscript will be scheduled for publication.

An invoice will be generated when your article is formally accepted. Please note, if your institution has a publishing partnership with PLOS and your article meets the relevant criteria, all or part of your publication costs will be covered. Please make sure your user information is up-to-date by logging into Editorial Manager at Editorial Manager® and clicking the ‘Update My Information' link at the top of the page. For questions related to billing, please contact billing support.

If your institution or institutions have a press office, please notify them about your upcoming paper to help maximize its impact. If they’ll be preparing press materials, please inform our press team as soon as possible -- no later than 48 hours after receiving the formal acceptance. Your manuscript will remain under strict press embargo until 2 pm Eastern Time on the date of publication. For more information, please contact onepress@plos.org.

Kind regards,

Kshitij Karki, MPH, MA

Academic Editor

PLOS One

Additional Editor Comments (optional):

Thank you for addressing the comments.

Reviewers' comments:

Reviewer's Responses to Questions

**Comments to the Author**

1. If the authors have adequately addressed your comments raised in a previous round of review and you feel that this manuscript is now acceptable for publication, you may indicate that here to bypass the “Comments to the Author” section, enter your conflict of interest statement in the “Confidential to Editor” section, and submit your "Accept" recommendation.

Reviewer #2: All comments have been addressed

**********

2. Is the manuscript technically sound, and do the data support the conclusions?

The manuscript must describe a technically sound piece of scientific research with data that supports the conclusions. Experiments must have been conducted rigorously, with appropriate controls, replication, and sample sizes. The conclusions must be drawn appropriately based on the data presented.

Reviewer #2: Yes

**********

3. Has the statistical analysis been performed appropriately and rigorously?

Reviewer #2: Yes

**********

4. Have the authors made all data underlying the findings in their manuscript fully available?

The PLOS Data policy requires authors to make all data underlying the findings described in their manuscript fully available without restriction, with rare exception (please refer to the Data Availability Statement in the manuscript PDF file). The data should be provided as part of the manuscript or its supporting information, or deposited to a public repository. For example, in addition to summary statistics, the data points behind means, medians and variance measures should be available. If there are restrictions on publicly sharing data—e.g. participant privacy or use of data from a third party—those must be specified.

Reviewer #2: Yes

**********

5. Is the manuscript presented in an intelligible fashion and written in standard English?

PLOS ONE does not copyedit accepted manuscripts, so the language in submitted articles must be clear, correct, and unambiguous. Any typographical or grammatical errors should be corrected at revision, so please note any specific errors here.

Reviewer #2: Yes

**********

6. Review Comments to the Author

Please use the space provided to explain your answers to the questions above. You may also include additional comments for the author, including concerns about dual publication, research ethics, or publication ethics. (Please upload your review as an attachment if it exceeds 20,000 characters)

Reviewer #2: I have no further comments. The authors have addressed my concerns satisfactorily, and I congratulate them on this work.

**********

7. PLOS authors have the option to publish the peer review history of their article (what does this mean?). If published, this will include your full peer review and any attached files.

If you choose “no”, your identity will remain anonymous but your review may still be made public.

**Do you want your identity to be public for this peer review?**  For information about this choice, including consent withdrawal, please see our Privacy Policy.

Reviewer #2: No

**********

# Editor report: Acceptance letter

**DOI:** 10.1371/journal.pone.0358730.r006

**Authors:** Kshitij Karki

PONE-D-26-00588R2

PLOS One

Dear Dr. Lwin,

I'm pleased to inform you that your manuscript has been deemed suitable for publication in PLOS One. Congratulations! Your manuscript is now being handed over to our production team.

At this stage, our production department will prepare your paper for publication. This includes ensuring the following:

* All references, tables, and figures are properly cited

* All relevant supporting information is included in the manuscript submission,

* There are no issues that prevent the paper from being properly typeset

You will receive further instructions from the production team, including instructions on how to review your proof when it is ready. Please keep in mind that we are working through a large volume of accepted articles, so please give us a few days to review your paper and let you know the next and final steps.

Lastly, if your institution or institutions have a press office, please let them know about your upcoming paper now to help maximize its impact. If they'll be preparing press materials, please inform our press team within the next 48 hours. Your manuscript will remain under strict press embargo until 2 pm Eastern Time on the date of publication. For more information, please contact onepress@plos.org.

You will receive an invoice from PLOS for your publication fee after your manuscript has reached the completed accept phase. If you receive an email requesting payment before acceptance or for any other service, this may be a phishing scheme. Learn how to identify phishing emails and protect your accounts at https://explore.plos.org/phishing.

If we can help with anything else, please email us at customercare@plos.org.

Thank you for submitting your work to PLOS One and supporting open access.

Kind regards,

PLOS One Editorial Office Staff

on behalf of

Dr. Kshitij Karki

Academic Editor

PLOS One
